# RAGER: A user-friendly computational platform for integrated analysis of RNA-Seq and ATAC-seq data

**DOI:** 10.1371/journal.pone.0349941

**Published:** 2026-05-22

**Authors:** Yunjie Liu, Yijia Liu, Zhouming Zhang, Fei Li, Hua Yu, Lu Lu

**Affiliations:** 1 Jiangxi Provincial Key Laboratory of Tumor Biology, School of Basic Medicine, Jiangxi Medical College, Nanchang University, China; 2 The First School of Clinical Medicine of Nanchang University, China; 3 Queen Mary School, Nanchang University, China; 4 Jiangxi Provincial Key Laboratory of Hematological Diseases, Department of Hematology, The First Affiliated Hospital, Jiangxi Medical College, Nanchang University, China; 5 The MOE Basic Research and Innovation Center for the Targeted Therapeutics of Solid Tumors, School of Basic Medicine; The First Affiliated Hospital, Jiangxi Medical College, Nanchang University, Nanchang, Jiangxi, China; 6 School of Intelligent Medicine and Information Engineering, Jiangxi University of Chinese Medicine, China; UC Los Angeles: University of California Los Angeles, UNITED STATES OF AMERICA

## Abstract

Technical advances in RNA sequencing (RNA-seq) and Assay for Transposase-Accessible Chromatin sequencing (ATAC-seq) provide us a genome-wide method to deeper insights into gene expression and regulation. The lack of user-friendly bioinformatics platforms poses a challenge for accurately interpreting such datasets. Here, we present a computational platform, RAGER, that integrates the popular bioinformatics tools in an automated thread for joint mining of RNA-seq and ATAC-seq data. RAGER facilitates integrative analysis of transcriptome and chromatin accessibility by providing an automated workflow that minimizes the need for bioinformatics expertise and significantly reduces processing time. We demonstrate RAGER’s utility for novel biological discovery by characterizing the transcriptome and chromatin accessibility of two recently published datasets. RAGER was implemented using Snakemake and is freely available via https://github.com/bioinfo202408/RAGER.

## Introduction

Next-generation sequencing (NGS) is a high-throughput sequencing technology that enables the simultaneous analysis of millions to billions of DNA or RNA fragments [1], including RNA sequencing (RNA-seq), Assay for Transposase-Accessible Chromatin using sequencing (ATAC-seq), etc [2,3]. NGS is increasingly applicable due to ongoing technological advancements and improved understanding. For example, mutation studies, cancer detection, disease diagnosis and therapy are the most studied topics [4,5].

RNA-Seq employs deep-sequencing technologies to convert RNA populations into cDNA libraries, which are then sequenced to produce short reads that can be aligned to a reference genome or assembled *de novo*, generating a comprehensive genome-scale transcription map [6]. In addition, ATAC-seq identifies accessible chromatin regions and transcription factor (TF) footprinting by utilizing the activity of a specially engineered, highly active Tn5 transposase that is equipped with sequencing adapters [3,7]. The synergistic analysis of RNA-seq and ATAC-seq data demonstrates significant value across multiple domains, including but not limited to ferroptosis [8], sex differentiation [9], cell aging [10] and cytokine activation [11]. However, integrating data from these two modalities and performing enrichment analysis present challenges, including: (1) low throughput, (2) time-consuming processes, and (3) reproducibility issues [12–14]. Therefore, there is a strong need for pipelines that can easily automate these steps.

To counteract these challenges, we introduce RAGER, an intuitive computational platform that integrates widely-used bioinformatics tools into an automated pipeline for the co-analysis of ATAC-seq and RNA-seq data. RAGER facilitates integrative transcriptome and chromatin accessibility analysis by offering a procedure that reduces processing time and necessitates little knowledge of bioinformatics. We assessed the value of RAGER using RNA-seq and ATAC-seq datasets generated from murine embryonic stem cells (GSE85632) and human CD34 + cells (GSE261119) [15,16].

In detail, RAGER identifies high-confidence regulatory gene circuits and discoveries novel biological knowledge by: 1) detecting differentially expressed genes (DEGs) from RNA-seq data (FDR < 0.05, |log2FC| > threshold) following rigorous quality control for read alignment assessment (>70% mapped reads) and 2) identifying genes associated with differential chromatin accessibility peaks (*q*-value < 0.05) at promoter/enhancer regions from ATAC-seq data. For genes showing concordant regulation at both promoter and enhancer regions, we generated: (i) expression profiles, (ii) chromatin accessibility patterns at regulatory elements, and (iii) quantitative scatter plots of expression-accessibility correlations. Subsequent functional characterization included Gene Set Enrichment Analysis (GSEA) to establish gene-function associations and TF binding motif analysis of regulatory regions to construct TF-gene or TF-enhancer regulatory networks. Notably, RAGER is extensible to user-provided gene sets beyond those identified through our integrative analysis.

### Implementation

**Fig 1:**
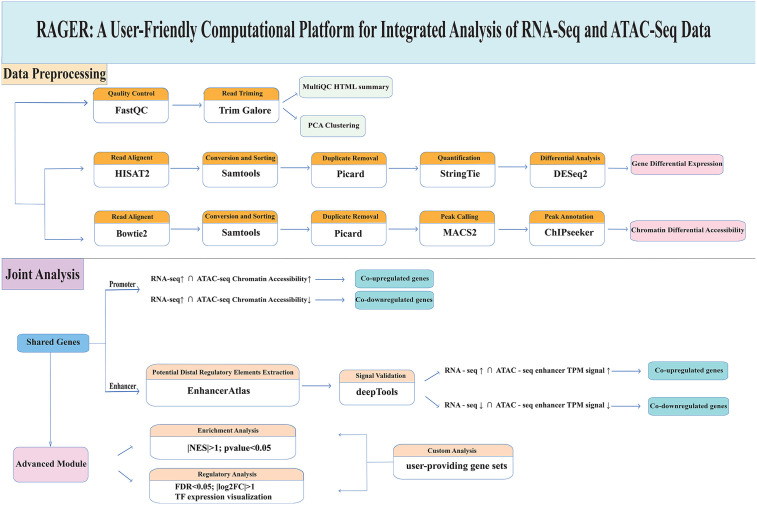
Schematic of the RAGER Pipeline for RNA-seq and ATAC-seq Data Visualization. The workflow initiates with the quality control and preprocessing of raw sequencing reads using FastQC, Trim Galore, and MultiQC. RNA-seq data are aligned with HISAT2, processed using Samtools and Picard, and quantified via StringTie. Differential gene expression analysis is carried out with DESeq2. ATAC-seq data are aligned using Bowtie2, similarly processed for duplicate removal, and peaks are identified with MACS2 and annotated through ChIPseeker. Joint analyses integrate promoter and enhancer regions to identify co-upregulated or co-downregulated genes based on consistent changes in gene expression and chromatin accessibility. Enhancer-associated signals are extracted from EnhancerAtlas and validated using deepTools. Advanced functional and regulatory analyses encompass enrichment analysis with clusterProfiler, transcription factor activity visualization, and support for user-provided gene sets in custom analyses.

The RAGER pipeline is implemented as a Snakemake workflow (v8.28.0), which dynamically links inputs to outputs through modular rules with dependency inference (Fig 1) [17]. This design facilitates automatic optimization of job execution order and resource allocation in cluster environments while ensuring reproducibility. The workflow begins with raw data processing where SRA files are converted to FASTQ format using fastq-dump tool for SRA-tools software (v3.2.0) (If you have your own sequencing data, skip this step). Quality control was performed using Trim Galore (v0.6.10) with parameters --phred33 -q 25 --length 35 --stringency 3 --paired --fastqc for paired end data to enforce Phred33 quality scoring (minimum Q25), remove adapters (3-base stringency), and discard reads below 35 bp, while generating per-sample FastQC reports. These reports were aggregated into a unified MultiQC HTML summary for systematic evaluation of raw data quality. Post-alignment QC included RNA-seq-specific analyses via RSeQC (v5.0.4, read coverage distribution curves and gene body coverage heatmaps) and ATAC-seq-specific assessments using ATACseqQC (v1.30.0, insert fragment size distribution, TSS enrichment profiles, and TSS enrichment heatmap, complemented by PCA and clustering plots for both data types to confirm reproducibility. RNA-seq reads are aligned using HISAT2 [18] (v2.1.1 with parameters --dta -S --summary-file for strand-specific mapping to Genome Reference), while ATAC-seq data is processed through Bowtie2 [19] (v2.5.4 -t -q -N 1 -L 25 --no-mixed --no-discordant) alignment, duplicate removal (Picard v2.18.2 MarkDuplicates), RNAseq expression was calculated by StringTie software(v3.0.0) using default parameters and ATACseq peak calling (MACS2 v2.2.9.1 with --keep-dup all -q 0.05). The pipeline architecture features dynamic job scheduling with automatic resource allocation and conditional recomputation where unchanged inputs bypass redundant processing steps. Configuration is managed through YAML files specifying reference genomes (GRCm38, GRCh38) and analysis parameters (e.g., FDR thresholds, log2FC thresholds). Output directories are systematically organized into ATACseq/, RNAseq/, Promoter_region_analysis/, Enhancer_region_analysis/, and Custom_genes_analysis/, subfolders containing BAM alignments, shared differential genes, and Cytoscape network of gene-function, TF-gene and TF-enhancer regulatory networks. To ensure reproducibility across HPC environments, the workflow is containerized, with all dependencies documented in a conda environment file (rager.yml). Furthermore, we have extended RAGER’s compatibility to support plant species with complex genomes. Additionally, the web resources of used tools are listed in Table 1.

**Table 1:** Overview of Tools and Resources Utilized in the RAGER Pipeline for ATAC-seq and RNA-seq Data Analysis.

Tool and resource	Version	Download URL
Snakemake	v8.28.0	https://snakemake.readthedocs.io/en/latest/index.html
Sra-tools	v3.2.0	https://github.com/ncbi/sra-tools
Trim Galore	v0.6.10	https://github.com/FredrikNorden/trim_galore
RSeQC	V5.0.4	https://bioconductor.org/packages/release/bioc/html/RSeQC.html
ATACseqQC	v1.30.0	https://jianhong.github.io/ATACseqQC/articles/ATACseqQC.html
HISAT2	v2.1.1	http://daehwankimlab.github.io/hisat2/
Bowtie2	v2.5.4	https://bowtie-bio.sourceforge.net/bowtie2/index.shtml
Samtools	v1.21	https://www.htslib.org/
Picard	v2.20.4	https://broadinstitute.github.io/picard/
StringTie	V3.0.0	https://ccb.jhu.edu/software/stringtie/
MACS2	v2.2.9.1	https://pypi.org/project/MACS2/
DESeq2	v1.46.0	https://www.rdocumentation.org/packages/DESeq2/
ChIPseeker	v1.40.0	https://rdrr.io/github/GuangchuangYu/ChIPseeker/man/ChIPseeker-package.html
deepTools	v3.5.6	https://deeptools.readthedocs.io/en/develop/
clusterProfiler	v4.14.0	https://bioc.r-universe.dev/clusterProfiler
Cytoscape	v3.10.3	https://cytoscape.org/
MEME Suite (AME)	v5.5.7	https://meme-suite.org/meme/meme_5.5.7/
IGV (Integrative Genomics Viewer)	v2.19.3	https://igv.org/
TxDb.Hsapiens.UCSC.hg38.knownGene	v3.20.2	https://www.bioconductor.org/packages/release/data/annotation/html/TxDb.Hsapiens.UCSC.hg38.knownGene.html
org.Hs.e.g.,db	v3.20.0	https://www.bioconductor.org/packages/release/data/annotation/html/org.Hs.e.g.,db.html
BSgenome.Hsapiens.UCSC.hg38	v1.4.5	https://bioconductor.org/packages/release/data/annotation/html/TxDb.Mmusculus.UCSC.mm10.knownGene.html
TxDb.Mmusculus.UCSC.mm10.knownGene	v3.10.0	https://www.bioconductor.org/packages/release/data/annotation/html/TxDb.Mmusculus.UCSC.mm10.knownGene.html
org.Mm.e.g.,db	v3.20.0	https://www.bioconductor.org/packages/release/data/annotation/html/org.Mm.e.g.,db.html
BSgenome.Mmusculus.UCSC.mm10	v1.4.3	https://bioconductor.org/packages/release/data/annotation/html/BSgenome.Mmusculus.UCSC.mm10.html
ggplot2	v3.5.2	http://cran.r-project.org/web/packages/ggplot2/index.html
tidyr	v1.3.1	https://cran.r-project.org/web/packages/tidyr/index.html
pheatmap	v1.0.13	https://cran.r-project.org/web/packages/pheatmap/index.html
wesanderson	v0.3.7	https://cran.r-project.org/web/packages/wesanderson/index.html
dendextend	v1.19.1	https://cran.r-project.org/web/packages/dendextend/index.html
psych	v2.5.6	https://cran.r-project.org/web/packages/psych/index.html
scatterplot3d	v0.3	https://cran.r-project.org/web/packages/scatterplot3d/index.html
Rsamtools	v2.22.0	https://bioconductor.org/packages/release/bioc/html/Rsamtools.htmll
dplyr	v1.1.4	https://cran.r-project.org/web/packages/dplyr/index.html
ggrepel	v0.9.6	https://cran.r-project.org/web/packages/ggrepel/index.html
ggVennDiagram	v1.5.4	https://cran.r-project.org/web/packages/ggVennDiagram/index.html
cols4all	v0.8	https://cran.r-project.org/web/packages/cols4all/index.html
AnnotationDbi	v1.68.0	https://bioconductor.org/packages/release/bioc/html/AnnotationDbi.html
ComplexHeatmap	v2.22.0	https://www.bioconductor.org/packages/devel/bioc/html/ComplexHeatmap.html
circlize	v0.4.16	https://cran.r-project.org/web/packages/circlize/index.html
Matrix	v1.7	https://cran.r-project.org/web/packages/Matrix/index.html
igraph	v2.0.3	https://cran.r-project.org/web/packages/igraph/index.html
scales	v1.4.0	https://cran.r-project.org/web/packages/scales/index.html
enrichplot	v1.26.1	https://www.bioconductor.org/packages/release/bioc/html/enrichplot.html
viridis	v0.6.5	https://cran.r-project.org/web/packages/viridis/vignettes/intro-to-viridis.html
StreamLit	v1.52.2	https://github.com/streamlit/streamlit
GenomicFeatures	v1.58.0	https://github.com/Bioconductor/GenomicFeatures
GenomicRanges	v1.58.0	https://bioconductor.org/packages//release/bioc/html/GenomicRanges.html
rtracklayer	v1.66.0	https://bioconductor.org/packages//release/bioc/html/rtracklayer.html
txdbmaker	v1.2.0	https://github.com/Bioconductor/txdbmaker
annotationforge	v1.48.0	https://github.com/Bioconductor/AnnotationForge
BSgenome	v1.74.0	https://github.com/Bioconductor/BSgenome
BSgenomeForge	v1.6.0	https://github.com/Bioconductor/BSgenomeForge
Trinity	v2.15.2	https://github.com/trinityrnaseq/trinityrnaseq
BLAST	v2.16.0	https://blast.ncbi.nlm.nih.gov/Blast.cgi

### Differential analysis module

RNA-seq differential expression analysis was carried out through sequential processing steps beginning with raw read quality control and adapter trimming using Trim-galore (v0.6.10) with parameters --phred33 --quality 25 --length 35 --fastqc, followed by alignment to reference genome (GRCm38 or GRCh38) using HISAT2 (v2.1.1) with --dta -S --summary-file parameters for strand-specific mapping followed by PCR duplicate removal with Picard MarkDuplicates (v2.18.2). Transcript assembly and quantification was conducted with StringTie (v3.0.0) using default parameters, with subsequent RNA-seq quality assessment using RSeQC (v5.0.4) for comprehensive evaluation of sequencing quality. Differential expression analysis was ultimately performed with DESeq2 (v1.46.0) applying genes with FDR < 0.05 and absolute log2 fold change > threshold as statistically significant. For ATAC-seq data analysis, reads were aligned using Bowtie2 (v2.5.4) with -t -q -N 1 -L 25 --no-mixed --no-discordant parameters, followed by PCR duplicate removal with Picard MarkDuplicates (v2.18.2) and data quality assessment using ATACseqQC (v1.30.0). Peak calling was performed with MACS2 (v2.2.9.1) using --keep-dup all -q 0.05 parameters, with subsequent peak annotation conducted using ChIPseeker (v1.42.0). We systematically analyzed promoter-associated differential genes by intersecting RNA-seq upregulated genes with genes linked to ATAC-seq upregulated peaks for co-upregulated genes, and RNA-seq downregulated genes with genes associated with ATAC-seq downregulated peaks for co- downregulated genes. For enhancer analysis, we first identified upregulated and downregulated genes at the transcriptional level and then extracted their potential distal regulatory elements using EnhancerAtlas (v2.0) within 1MB genomic windows. These enhancers were further validated through ATAC-seq signal profiling using deepTools (v3.5.6) computeMatrix in reference-point mode (-b 1000 -a 1000 --binSize 10 parameters). Enhancer-gene regulatory relationships were defined as follows: Enhancers exhibiting a |log2FC| > 1 in the experimental versus control group were considered differentially accessible. Enhancers with log2FC > 1 were classified as co-upregulated enhancers, and their associated genes were designated co-upregulated genes. Conversely, enhancers with log2FC < −1 were classified as co-downregulated enhancers, with their associated genes designated co-downregulated genes. In addition, the resulting differential genes/peaks from both promoter and enhancer analyses were subsequently used for functional enrichment and regulatory network construction.

### Enrichment analysis module

GSEA was performed using the clusterProfiler package (v4.14.0) against KEGG and GO gene set collections. Analyses employed 1,000 phenotype permutations and weighted scoring, with significant pathways requiring normalized enrichment score |NES| > 1 and pvalue < 0.05. Resulting gene-function networks were visualized in Cytoscape (v3.10.3) (node weighting by NES and log2FoldChange).

### Regulatory analysis module

Transcription factor binding motif analysis was performed using MEME Suite (v5.5.7) with the “AME” program, leveraging the JASPAR2024 CORE vertebrate database. Motif enrichment analysis was performed using AME with default parameters to identify known database motifs enriched in promoter sequences of shared genes and in shared enhancer sequences, which employs position-specific scoring matrices and calculates enrichment significance through Fisher’s exact tests followed by Benjamini-Hochberg correction (FDR < 0.05). Position weight matrices of significantly enriched motifs (FDR < 0.05) and corresponding transcription factors were annotated using Mus_musculus_TF and Homo_sapiens_TF from AnimalTFDB4. The expressions of enriched TFs were visualized by heatmap using a custom R script. In addition, we also checked the peak signal in the gene browser (IGV v2.19.3). Subsequently, we constructed the TF-Gene and TF-Enhancer regulatory networks, which were displayed using Cytoscape software (node weighting by PWM-score and -log10pvalue).

### Custom Analysis Module

For user-provided gene sets, the pipeline activates alternative analysis branches including GSEA analysis, and transcription factor binding motif analysis (using MEME Suite AME tool). Results were generated as publication-ready figures (PDF) and machine-readable data tables (CSV).

### Local UI Module

A local graphical user interface (GUI) was developed using Streamlit (v1.52.2) to facilitate pipeline execution and improve accessibility for users without extensive Linux command-line experience. The UI provides four modules, and it is recommended to run them in the following order: (1) Preprocess RNAseq (2) Preprocess ATACseq (3) Joint analysis (4) Custom analysis (S12 Fig). This design enables users to complete end-to-end analyses through an interactive workflow with simplified parameter configuration and standardized output generation.

### Unmapped Reads Module

To enhance the applicability of RAGER to species with incomplete or fragmented reference genomes, we added an optional module for analyzing unaligned RNA-seq reads and unaligned ATAC-seq reads. For RNA-seq, unaligned reads were extracted from the original SAM/BAM files using samtools (v1.21). *De novo* transcriptome assembly was then performed on paired unaligned reads using Trinity software (v2.15.2). The assembled contigs were further annotated by performing sequence similarity searches against reference complementary DNA and protein databases using BLAST (v2.16.0) for both nucleotide and protein sequence alignments. For each contig, the best matching sequence was selected based on bit score and expectation value.

We also developed an optional module for analyzing unaligned ATAC-seq reads. Unaligned reads were extracted from the original SAM/BAM alignment files using samtools (v1.21) and converted to FASTA format for sequence classification. These reads were then realigned against chloroplast and mitochondrial genome references using Bowtie2 in single-end mode. Reads that remained unassigned after organelle filtering were subsequently aligned against repetitive element/transposable element (TE) reference sequences. Reads not classified into any of the above reference categories were retained as unclassified fragments. For each sample, the number and proportion of reads assigned to chloroplast, mitochondrial, repetitive element/TE, and unclassified categories were calculated. This module aims to facilitate straightforward interpretation of unaligned RNA-seq and ATAC-seq reads and enhance the utility of RAGER in species with incomplete reference genome assemblies. Both modules are designed as optional extensions and do not alter the reference genome-guided main pipeline.

## Results

To demonstrate RAGER’s capabilities, we performed two case studies using matched RNA-seq and ATAC-seq datasets from mouse (GSE85632) and human (GSE261119). The mouse dataset comprised 16 paired-end fastq files (SRR4032350-SRR4032353, SRR4032269-SRR4032272), while human data included 16 paired-end fastq files (SRR28263042-SRR28263045, SRR28263046-SRR28263049).

### Applying RAGER to GSE85632

Raw sequencing data were downloaded from the GEO database (GSE85632) [20]. From this dataset, we selected specific SRR files (SRR4032350, SRR4032351, SRR4032352, SRR4032353, SRR4032269, SRR4032270, SRR4032271, SRR4032272) relevant to our analysis, resulting in a total of 16 fastq files. The original study demonstrated that murine DUX and its human ortholog DUX4 function as key transcription factors, activating cleavage-stage-specific genes (e.g., ZSCAN4, KDM4E) and endogenous retroviral elements (MERVL/HERVL) in mouse embryonic stem cells (mESCs). Ectopic expression of DUX in mESCs induced a two-cell embryo-like (2C-like) state, characterized by transcriptional reprogramming, dissolution of POU5F1 protein networks, and chromatin remodeling as measured by ATAC-seq. These findings established DUX/DUX4 as conserved regulators of early embryonic transcriptional programs. We selected this dataset for our analysis due to its matched RNA-seq and ATAC-seq profiles, high-quality replicates, and well-defined regulatory dynamics. These characteristics make it an ideal benchmark for evaluating RAGER’s performance in detecting differential gene expression, chromatin accessibility changes, and reconstructing regulatory networks. Our reanalysis applied updated bioinformatics standards, including alignment to the GRCm38 reference genome and stringent quality control metrics and Identification of common genes with significant biological implications, ensuring robust and reproducible results.

### Alignment rate statistics, differential gene acquisition, and differential analysis with RAGER

**Fig 2:**
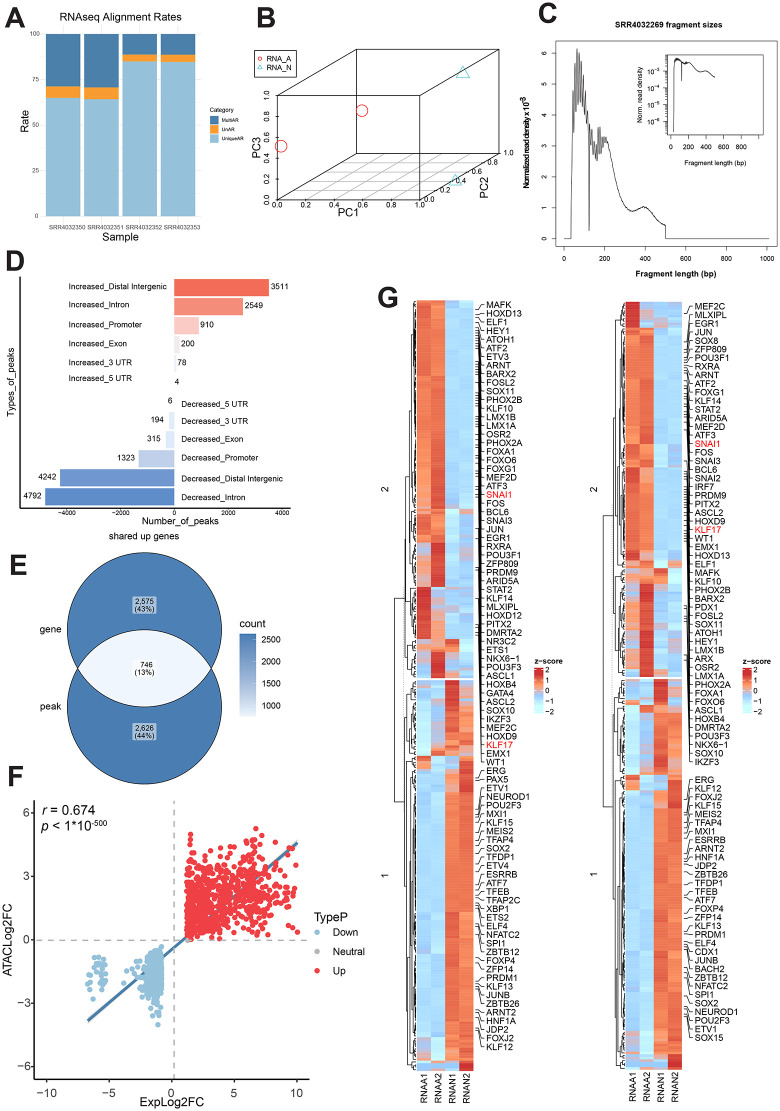
Application of the RAGER to the GSE85632 reveals coordinated gene regulatory mechanisms. **(A)** Bar plot showing RNA-seq read alignment rates for all samples in the GSE85632. **(B)** Three-dimensional Principal Component Analysis (3D PCA) plot of the RNA-seq data, showing the transcriptomic profiles of each sample. **(C)** Insert size distribution of ATAC-seq fragments for a representative sample (SRR4032269) from the first dataset. **(D)** Genomic distribution of ATAC-seq peaks that were significantly increased (Up) or decreased (Down). **(E)** Venn diagram showing the overlap between genes with significantly up-regulated expression (RNA-seq, log2FC > 1, p-value < 0.05) and genes associated with up-regulated ATAC-seq peaks. **(F)** Scatter plot of log2 fold-change (log2FC) values for the overlapping genes identified in **(E)**. **(G)** Heatmaps showing the RNA-seq expression levels of transcription factors (TFs) whose binding motifs are significantly enriched in the promoter regions of shared genes. (The left panel corresponds to TFs enriched in co-upregulated genes, and the right panel corresponds to TFs enriched in co-downregulated genes.).

RNA-seq alignment quality assessment (Fig 2A) demonstrated high mapping efficiency across all samples, with unique alignment rates (UniqueAR) exceeding 60% (range: 60–84%). Multi-mapped reads (MultiAR) accounted for 11.3–29.3% of total reads, while unmapped rates (UnAR) remained below 6.5%, meeting ENCODE quality thresholds for downstream analysis. PCA (Fig 2B) revealed clear separation between RNA_A (activated state induced by DUX4/Dux) and RNA_N (control) groups along PC1, with tight clustering of biological replicates within each group. The effects achieved by quality trimming our data of RNA-seq were shown in S1 Fig.

ATAC-seq data fragment size distribution was shown in (Fig 2C), exhibiting the data of SRR4032269 concentrated around 100–150 bp. As demonstrated in (Fig 2D), ATAC-seq peak annotation statistics plot reveals increased accessibility in intergenic (3,511 peaks) and intronic regions (2,549 peaks), suggesting activation of distal regulatory elements, while decreased accessibility predominates in introns (4,792 peaks) and intergenic regions (4,242 peaks), indicating large-scale silencing. Promoter accessibility gains (910 peaks) correlate with target gene activation. The quality control results of our ATAC-seq raw data and the results of after alignment are presented in S2 Fig.

The genomic annotation of ATAC-seq peaks and identification of shared genes were displayed in S3 Fig. Venn analysis (Fig 2E; S3 Fig) revealed 746 co-upregulated genes (shared between RNA-seq up-DEGs and ATAC-seq promoter-open peaks) and 707 co-downregulated genes (shared between RNA-seq down-DEGs and promoter-closed peaks) (FDR < 0.05 for both omics layers). The expression-accessibility correlation scatterplot (Fig 2F) demonstrated an overall strong positive correlation (*r* = 0.674, *p* < 1*10^-500^), primarily driven by upregulated genes, while a weaker positive correlation was observed for downregulated genes.

### The biological validation of RAGER against the original research findings

Heatmaps (Fig 2G) displayed RNA-seq expression levels of TFs with binding motifs significantly enriched in the promoters of shared genes, in which KLF17 and SNAI1—key factors from the original study—are highlighted in red. S4 Fig illustrated significant signaling pathways associated with embryonic development, meeting the criteria of |NES| > 1 and p-value < 0.05. For example, translational control mediated by initiation factor activity plays pivotal roles in early embryonic development by orchestrating multiple fundamental processes including the timed activation of maternal mRNAs, the precise regulation of protein synthesis rates during rapid cleavage divisions and the establishment of embryonic polarity [21–24]. In S4E Fig, genes regulated by co-upregulated enhancers were displayed, showing their NES and corresponding significance levels. This panel highlighted biological processes and molecular functions that are potentially enhanced through these genes, contributing to the overall understanding of the regulatory mechanisms under study. S5B Fig displayed the 5 significant TFs in the upregulated promoter regions which are in agreement with the original study, as well as the 10 genes that each of these TFs regulates the most strongly, where gene size represents enrichment score and TF color indicates statistical significance. Validation of RAGER confirmed the concordant regulation at RNA and chromatin levels (S5C Fig), including the representative factors KLF17.

In their seminal work, Hendrickson et al. established comprehensive RNA-seq and ATAC-seq reference maps of human pre-implantation development, highlighting DUX4’s central role in activating cleavage-stage genes and retrotransposons. Although their study identified KLF17 and SNAI1 as transcription factors directly bound by DUX4, the functional significance of these factors within the DUX4 regulatory network was not investigated. Our RAGER-based reanalysis not only validated KLF17 and SNAI1 (Fig 2G; S5 Fig) as integral components of the DUX4 regulatory program but also uncovered their divergent chromatin accessibility signatures during embryonic genome activation. Notably, while maternal KLF17 serves as a transcriptional activator of zygotic genome activation in mouse embryos through RNA Polymerase II recruitment [25]. These findings position RAGER as a powerful tool for uncovering hidden regulatory architectures in developmental systems. In addition, our approach has exposed previously unrecognized layers of the DUX4 transcriptional hierarchy that may guide future studies of human embryogenesis and cellular reprogramming.

### Applying RAGER to GSE261119

Raw sequencing data were downloaded from the GEO database (GSE261119) [16], which includes RNA-seq profiles of human hematopoietic stem and progenitor cells (HSPCs) under three experimental conditions: untreated controls, BRAF inhibitors-treated cells, and BRAF^V600E^-overexpressing cells. From this dataset, we specifically selected the GDC-0879-treated (a BRAF inhibitor small molecule) samples for analysis due to their unique capacity to promote transcriptional activation through paradoxical MAPK pathway stimulation. The original study demonstrated that GDC-0879 treatment amplifies ERK/MAPK signaling in wild-type HSPCs, leading to enhanced erythroid progenitor proliferation while temporarily delaying differentiation, a process accompanied by upregulation of cell cycle regulators like MYC and CDK6. These transcriptional changes were shown to depend on RAF dimerization and cytokine-mediated RAS activation, creating an ideal system for identifying enhancer-regulated genes associated with proliferative responses. Our reanalysis applied current bioinformatics standards, including alignment to the GRCh38 reference genome and stringent quality thresholds. These parameters ensure reliable detection of differentially expressed genes while maintaining compatibility with downstream enhancer prediction analyses through integration with publicly available chromatin accessibility data from related studies of human erythropoiesis.

### Alignment rate statistics, differential gene acquisition, and differential analysis with RAGER

**Fig 3:**
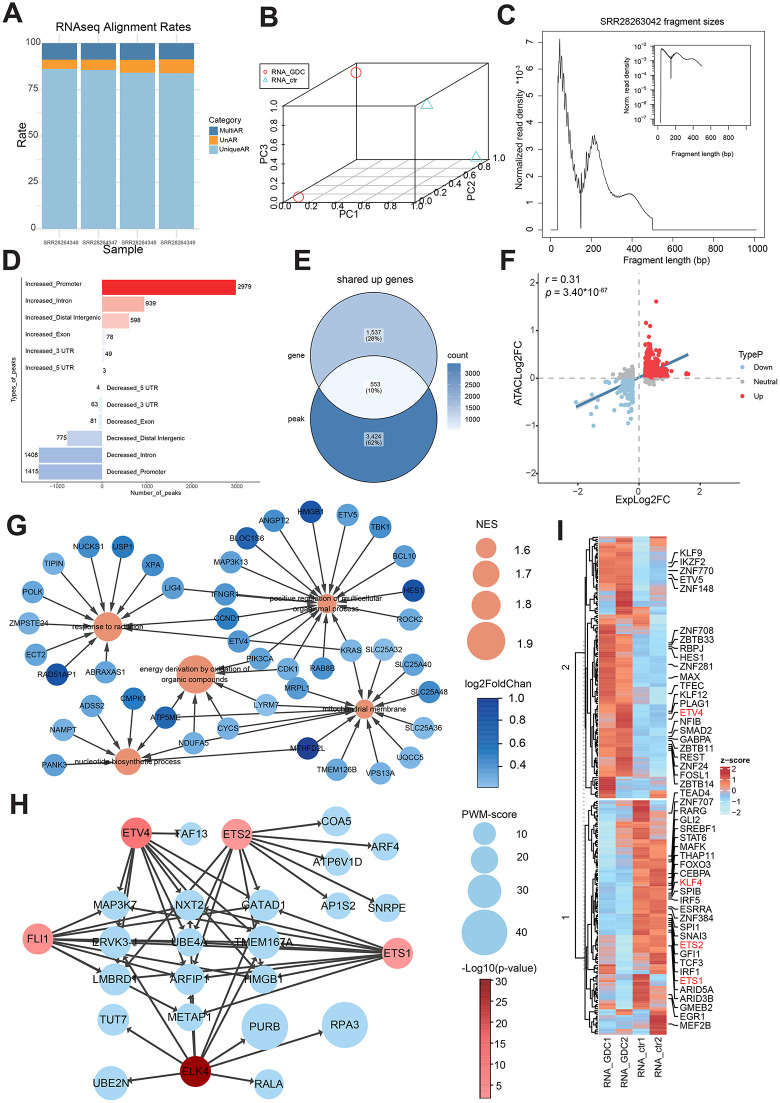
RAGER analysis of GSE261119 uncovers orchestrated gene regulatory programs. **(A)** Bar plot showing RNA-seq read alignment rates for all samples in the second dataset. **(B)** Three-dimensional Principal Component Analysis (3D PCA) plot of the RNA-seq data, showing the transcriptomic profiles of each sample. **(C)**Insert size distribution of ATAC-seq fragments for a representative sample (SRR28263042) from the GSE261119. **(D)** Genomic distribution of ATAC-seq peaks that were significantly increased (Up) or decreased (Down). **(E)** Venn diagram showing the overlap between genes with significantly up-regulated expression (RNA-seq, log2FC > 0.2, p-value < 0.05) and genes associated with up-regulated ATAC-seq peaks. **(F)** Scatter plot of log2 fold-change (log2FC) values for the overlapping genes identified in **(E)**. **(G)** Cytoscape network graph of Gene Ontology (GO) terms significantly enriched for co-upregulated genes linked to promoter regions. **(H)** Cytoscape network graph depicting the top 10 target genes (ranked by enrichment score) for each transcription factor (TF), with regulatory relationships validated by the original research.(TFs were significantly enriched to shared up-regulated genes associated with promoter regions.). **(I)** Heatmap of RNA-seq expression levels for transcription factors (TFs) whose binding motifs are significantly enriched in the promoter regions of the co-upregulated genes.

The alignment quality assessment demonstrated consistently high performance across all samples (Fig 3A). Between 83.8–86.1% of reads showed UniqueAR, while MultiAR account for less than 9% of total sequences. The UnAR remained within an acceptable 5–7.5% range, comfortably meeting ENCODE quality standards for subsequent analyses. PCA of transcriptomic profiles revealed that PC1, explaining the majority of variance, clearly separated GDC-0879-treated samples (RNA_GDC, PC1 < 0.2) from control samples (RNA_ctr, PC1 ≈ 1.0) along the horizontal axis (Fig 3B). In contrast, PC2 showed minimal separation between groups (PC2 ≈ 0.6–1.0), indicating the treatment effect dominated the transcriptional variance. Our RNA-seq data’s quality trimming results were illustrated in S6 Fig.

ATAC-seq data fragment size distribution analysis (Fig 3C) indicated a predominant 40–100 bp peak for SRR28263042. S7 Fig displayed the results of quality trimming our ATAC-seq data.

Chromatin accessibility profiling uncovered extensive remodeling induced by GDC-0879 treatment, with promoter regions exhibiting the most dramatic changes (2,979 increased accessibility peaks, Fig 3D). Intronic regions ranked second (939 peaks), followed by distal intergenic regions (598 peaks). More modest alterations appeared in exonic regions (78 peaks), 3’UTRs (49 peaks), and 5’UTRs (3 peaks).

S8 Fig showed the genomic annotation of ATAC-seq peaks and the discovery of shared genes. Venn diagram analysis (Fig 3E; S8 Fig) identified 553 concordantly upregulated genes showing both transcriptional activation and chromatin accessibility opening, alongside 258 downregulated genes associated with chromatin accessibility closing. The expression-accessibility correlation analysis (Fig 3F) revealed a robust positive correlation for upregulated genes (*r* = 0.31, *p* = 3.40*10^-67^), whereas downregulated genes exhibited a weaker positive correlation.

### The biological resonance of RAGER with the original research findings

For GSEA network diagram of co-upregulated genes (Fig 3G), we chose the most important pathways related with erythropoiesis, such as response to radiation, positive regulation of multicellular organismal process, energy derivation by oxidation organic compounds, mitochondrial membrane and nucleotide biosynthetic process. S9 Fig provided illustrations of additional significant signaling pathways through the GSEA analysis. For instance, by coordinating several basic processes, such as rapid proliferation and differentiation of erythroid progenitors, DNA synthesis during erythroblast maturation, and energy provision for hemoglobin production, plays an important part in erythropoiesis [26–28].

Fig 3H displayed the 5 significant TFs (ETV4, ETS2, FLI1, ETS1, and ELK4) which are in agreement with the original study, as well as the 10 genes that each of these TFs regulates the most strongly, where gene size represents enrichment score and TF color indicates statistical significance. The concordant regulation at the RNA and chromatin levels, such as the TF ETV4 (up) and ETS1 (down), was validated by RAGER (S10 Fig).

Our comprehensive TF heatmap analysis (Fig 3I; S10 Fig) systematically validated the TFs highlighted in Wu et al.’s original study through red-label marking, demonstrating complete concordance between our conjoint analysis and their chromatin-based evidence. The consistent identification of these factors through both transcriptional (our heatmap) and chromatin accessibility (Wu’s motif analysis) approaches reinforces their biological significance in MAPK-mediated erythropoiesis regulation as established in the original study.

### Applying RAGER to the Custom Analysis

To further demonstrate the flexibility and utility of RAGER for hypothesis-driven research, we applied the Custom Analysis Module to a user-provided gene set derived from the original study of GSE261119 [16]. We focused on a curated list of 92 differentially expressed genes implicated in erythropoiesis and the MAPK signaling pathway (extended from supplementary materials in Wu et al.), which included key regulators such as MYC, CDK6, BCL2L1, and GATA1.

RAGER’s Custom Analysis Module automatically processed this gene set through its alternative branches (Methods). Functional enrichment analysis via GSEA (S11A-B Fig) recapitulated the core biological themes of the original study, revealing significant enrichment (|NES| > 1, p-value < 0.05) for pathways.

TF binding motif analysis of the promoters of these custom genes using MEME Suite’s AME tool identified ETS1, ETV4 and KLF4 as the top significantly enriched TFs (S11D Fig). This result is highly consistent with the established regulatory paradigm in hematopoiesis, where KLF4 and ETS family factors coordinately regulate erythropoietic gene expression [29–31]. The expression levels of these enriched TFs, as measured by RNA-seq in the GSE261119 dataset, were visualized in a heatmap (S11C Fig), showing clear upregulation of ETV4, and downregulation of ETS1 and KLF4 in the GDC-0879 treated group.

This custom analysis underscores RAGER’s capability to seamlessly integrate external, biologically relevant gene sets into its analytical framework. The pipeline not only recapitulated the central findings of the source publication but also extended the analysis by identifying the master transcriptional regulators underlying the observed erythropoietic phenotype, thereby providing a mechanistic link between the MAPK pathway activation and the erythroid differentiation program. This functionality makes RAGER particularly valuable for researchers seeking to validate and explore specific hypotheses using their own gene lists of interest within a robust and reproducible bioinformatics environment.

### Applying RAGER to the Unmapped Reads

To evaluate the effectiveness of the optional modules for analyzing unaligned reads, we applied them to a representative RNA-seq samples (SRR21931110, SRR21931111) and ATAC-seq sample (SRR21931115, SRR21931116) from the public dataset GSE214739. The results are summarized in Table 2 and Table 3.

**Table 2:** Assembly and annotation summary of unmapped reads in the RNA-seq data from GSE214739.

Sample	Contigs (n)	N50 (bp)	Total assembled length (bp)	Annotated by BLASTN (n)	Annotated by BLASTX (n)	Overall Annotation rate (%)
SRR21931110	34,282	623	17,732,228	28,376	24,557	85.60
SRR21931111	49,833	511	22,646,463	42,801	34,901	87.85

Unmapped reads from the RNA-seq data were extracted from the alignment file and de novo assembled using Trinity. Assembly quality was summarized by the number of contigs, N50, and total assembled length. Functional annotation was evaluated by sequence similarity searches against transcript and protein reference databases using BLASTN and BLASTX, respectively. Overall annotation rate was calculated as the proportion of assembled contigs with at least one significant hit in either BLASTN or BLASTX.

**Table 3:** Source classification of unmapped reads in the ATAC-seq data from GSE214739.

Sample	Total unmapped reads (n)	cpDNA, [n(%)]	mtDNA, [n(%)]	TE, [n(%)]	Unclassified, [n(%)]
SRR21931115	67,121,658	1,959,166 (2.92)	3,461,439 (5.16)	10,221,985 (15.23)	51,479,068 (76.69)
SRR21931116	117,027,610	1,437,856 (1.23)	2,726,558 (2.33)	9,287,785 (7.94)	103,575,411 (88.50)

Unmapped reads from the ATAC-seq data were sequentially classified by alignment to chloroplast DNA (cpDNA), mitochondrial DNA (mtDNA), and transposable element/repetitive sequence (TE) references. Reads that could not be assigned to any of these categories were designated as unclassified. Values are shown as read counts and percentages relative to the total number of unmapped reads.

As shown in Table 2, a total of 34,282 contigs were assembled from the unaligned RNA-seq reads, with an N50 of 623 bp and a total assembled length of 17,732,228 bp. Among these contigs, 28,376 were successfully annotated by BLASTN against the reference transcript database, and 24,557 were annotated by BLASTX against the reference protein database. The overall annotation rate, defined as the proportion of contigs with at least one significant hit in either BLASTN or BLASTX, reached 85.6%. These results demonstrate that a substantial proportion of unaligned RNA-seq reads originate from transcripts not represented in the reference genome or annotation, and can be effectively recovered and functionally annotated through de novo assembly and sequence similarity searching.

Table 3 presents the classification results for unaligned ATAC-seq reads. Among the 67,121,658 unaligned reads, 1,959,166 (2.92%) were mapped to chloroplast DNA, 3,461,439 (5.16%) to mitochondrial DNA, and 10,221,985 (15.23%) to transposable element/repetitive sequence references. The remaining 51,479,068 reads (76.69%) could not be assigned to any of these categories and were therefore classified as unclassified fragments. These classifications help distinguish reads originating from organellar genomes or repetitive elements—which may represent biological signals rather than technical noise—from those that remain unassignable, thereby improving the interpretability of unaligned fragments in species with incomplete reference genomes.

Collectively, these analyses confirm that the optional modules effectively recover biologically meaningful information from unaligned reads across both transcriptomic and epigenomic data types, supporting the utility of RAGER in non-model organisms or species with lower-quality genome assemblies.

## Discussion

The RAGER pipeline successfully validated and extended key findings from both the mouse embryonic stem cells and human CD34 ＋ cells through integrated RNA-seq and ATAC-seq analysis. For the mouse data, our reanalysis not only confirmed KLF17 and SNAI1 as direct DUX4 targets but additionally demonstrated their functional relevance via correlated transcriptional upregulation and chromatin accessibility changes, with KLF17 showing stronger promoter accessibility while SNAI1 exhibited more distal regulatory element activation. In the human BRAF inhibitor study, RAGER provided comprehensive validation of the reported ETS-family transcription factors (ETS1/2, FLI1, ELK4, ETV4/5) through both transcriptional regulation and corresponding chromatin accessibility changes, while also identifying KLF4 as a consistently downregulated factor that may contribute to the observed differentiation delay. Using human datasets, the pipeline’s technical performance was also evidenced by its ability to identify 746 co-upregulated and 707 co-downregulated gene-region pairs while establishing significant expression-accessibility correlations (*r* = 0.674, *p* < 1*10^-500^), confirming its utility for the integration of RNA-seq and ATAC-seq without requiring additional experimental validation beyond the original studies. A current limitation of RAGER is the requirement for matched RNA-seq and ATAC-seq samples from the same biological system. These results demonstrate RAGER’s effectiveness for cross-species regulatory analysis while remaining strictly grounded in the experimental evidence presented in both source studies.

RAGER’s use in these research shows how versatile the methodology is for analyzing RNA-seq or ATAC-seq experiments respectively. RAGER’s performance was further contextualized through comparison with existing bioinformatics pipelines specializing in epigenomics and multi-omics integration (Table 4). Unlike DiffBind and esATAC [32], which focus primarily on ChIP-seq or ATAC-seq peak calling and differential binding, RAGER provides a unified environment for joint RNA-seq and ATAC-seq analysis, including co-differential gene identification, expression-accessibility correlation, and regulatory network reconstruction. While PRADA [33] and TRAPLINE [34] offer user-friendly interfaces and report generation, they lack built-in support for dual-omics and motif-TF regulatory analysis. Similarly, CoBRA [35]—though also Snakemake-based and containerized—does not explicitly incorporate enhancer-gene association or provide automated functional enrichment visualization. These features position RAGER as a uniquely holistic and biologist-friendly platform for joint analysis.

**Table 4:** Comparative Features of Bioinformatics Tools for Epigenomics Data Analysis.

Classification	Trait	DiffBind	PRADA	TRAPLINE	esATAC	CoBRA	RAGER
**Basic Information**	**Year**	2011	2014	2016	2018	2021	2025
	**Reference or Web Link**	https://bioconductor.org/packages/release/bioc/html/DiffBind.html	[33]	[34]	[32]	[35]	https://github.com/bioinfo202408/RAGER
	**Workflow Engine**	–	–	Galaxy	–	Snakemake	Snakemake
	**Containerization Support**	–	–	–	–	✓	✓
**Data Preprocessing Module**	**Standard Quality Control and Comparison**	✓	✓	✓	✓	–	✓
	**Peak Calling**	✓	–	–	✓	✓	✓
**Advanced Module**	**Joint Analysis**	–	–	–	–	✓	✓
	**Custom Analysis**	–	–	–	✓	✓	✓
	**Motif Enrichment**	✓	–	–	✓	✓	✓
	**Differential Analysis**	✓	✓	✓	–	–	✓
	**Chromatin State Enrichment**	✓	–	–	✓	✓	✓
**User Experience**	**One-click Report Generation**	–	✓	✓	–	–	✓
	**Detailed instruction with case studies**	–	✓	✓	–	✓	✓

While RAGER provides a practical framework for joint RNA-seq and ATAC-seq analysis, we acknowledge a fundamental limitation in its current strategy for linking distal enhancers to their target genes. The present version employs a distance-based assignment, which, while computationally simple, does not account for the three-dimensional architecture of chromatin that physically mediates enhancer-promoter communication. This often leads to false-positive or false-negative associations, as linear genomic proximity is an imperfect proxy for functional interaction. To address this, a major direction for the future development of RAGER will be the integration of chromatin conformation capture data (e.g., Hi-C, HiChIP) to implement a more sophisticated and biologically accurate model for enhancer-gene association. Inspired by the groundbreaking Activity-by-Contact (ABC) model—which quantifies an enhancer’s regulatory potential as the product of its biochemical activity and its contact frequency with a promoter—we envision a future version of RAGER that calculates an “ABC-like” score for candidate enhancer-gene pairs [36]. This upgrade would allow RAGER to distinguish functional, loop-mediated interactions from mere genomic adjacency, thereby significantly improving the precision of reconstructed regulatory networks and providing deeper mechanistic insights into gene regulation from multi-omics data.

In conclusion, we present RAGER, a computational pipeline characterized by speed, efficiency, flexibility, and user-friendliness, positioning it as a powerful resource for life scientists. A key strength of RAGER is its development by biologists, enabling a broad range of valuable analyses while maintaining accessibility for users with minimal computational expertise. New RNA-seq and ATAC-seq tools continue to emerge, advancing transcriptome analysis capabilities. Furthermore, RAGER was crafted to incorporate core principles essential for the meaningful interpretation of RNA-seq and ATAC-seq datasets across species, such as mouse and human. Nonetheless, our hope is that RAGER serves as a foundational framework that others can build upon and enhance, ultimately advancing our collective capacity to derive insights from transcriptomic data and chromatin accessibility data.

## Supporting information

S1 Fig Quality control (QC) metrics for the RNA-seq data of the GSE85632. **(A)** Summary table of key RNA-seq QC metrics generated by MultiQC, including duplication rate (Dups), GC content, average and median read length, failure rate, and total number of sequences for each sample. **(B)** Principal Component Analysis (PCA) plots showing transcriptomic sample relationships and variance across three pairs of principal components: PC1 vs. PC2, PC1 vs. PC3, and PC2 vs. PC3. **(C)** Cluster dendrogram depicting the relatedness of samples based on their global gene expression profiles. **(D)** Gene body coverage plot (output by RSeQC), showing the normalized read coverage across the length of genes (from 5’ to 3’ end) for each sample, which assesses potential 5’ or 3’ bias. **(E)** Gene body coverage heatmap (output by RSeQC), providing an alternative visualization of the uniformity of read coverage across gene bodies for all samples.
(PDF)

S2 Fig Quality control (QC) metrics for the ATAC-seq data of the GSE85632. **(A)** Bar plot showing the ATAC-seq read alignment rates for all samples. **(B)** Summary table of key ATAC-seq QC metrics generated by MultiQC, including duplication rate (Dups), GC content, average and median read length, failure rate, and total number of sequences for each sample. **(C)** Three-dimensional Principal Component Analysis (3D PCA) plots showing epigenomic sample relationships and variance across pairs of principal components (PC1 vs. PC2, PC1 vs. PC3). **(D)** Cluster dendrogram depicting the relatedness of samples based on their chromatin accessibility profiles. **(E)** Transcription start site (TSS) enrichment plot (output by ATACseqQC) for sample SRR4032269, indicating the quality of the ATAC-seq library by the nucleosome-free signal at gene promoters. **(F)** Transcription start site (TSS) enrichment heatmap (output by ATACseqQC) for sample SRR4032269, providing an alternative visualization of the enrichment signal.
(PDF)

S3 Fig Genomic annotation of ATAC-seq peaks and identification of shared genes in the GSE85632. **(A)** Bar plot showing the total number of ATAC-seq peaks annotated to various genomic regions (e.g., promoter, enhancer, intron, intergenic). **(B)** Venn diagram showing the overlap between genes with significantly down-regulated expression (RNA-seq, log2FC < −1, p-value < 0.05) and genes associated with significantly down-regulated ATAC-seq peaks. **(C)** Volcano plot displaying the differential expression of all the shared genes associated with promoter region. Significantly co-upregulated and co-downregulated genes are highlighted. **(D)** Volcano plot displaying the differential expression of all the shared genes associated with enhancer region. Significantly co-upregulated and co-downregulated genes are highlighted.
(PDF)

S4 Fig Gene Set Enrichment Analysis (GSEA) of shared genes in the GSE85632. **(A, B)** GSEA result of Gene Ontology (GO) biological pathways significantly enriched (|NES| > 1, p-value < 0.05) for co-upregulated and co-downregulated genes associated with promoter regions, respectively. **(C, D)** GSEA result of GO biological pathways significantly enriched (|NES| > 1, p-value < 0.05) for co-upregulated and co-downregulated genes associated with enhancer regions, respectively. **(E)** Cytoscape network visualization of GO biological pathways significantly enriched (|NES| > 1, p-value < 0.05) for the shared up-regulated genes associated with enhancer regions.
(PDF)

S5 Fig Transcription factor (TF) analysis and validation of key regulators in the GSE85632. **(A)** Heatmap of RNA-seq expression levels for TFs whose binding motifs are significantly enriched in the co-upregulated and co-downregulated enhancer regions. Genes related to the original research are highlighted. **(B)** Cytoscape network graph depicting the top 10 target genes (ranked by enrichment score) for each transcription factor (TF), with regulatory relationships validated by the original research (TFs were significantly enriched to shared up-regulated genes associated with promoter regions). **(C)** Integrative Genomics Viewer (IGV) browser tracks showing increased chromatin accessibility (ATAC-seq) at the genomic locus of KLF17, which is also up-regulated at the RNA-seq level.
(PDF)

S6 Fig Quality control (QC) metrics for the RNA-seq data of the GSE261119. **(A)** Summary table of key QC metrics generated by MultiQC, including duplication rate (Dups), GC content, average and median read length, failure rate, and total number of sequences for each sample. **(B)** Principal Component Analysis (PCA) plots showing sample relationships and variance across three pairs of principal components: PC1 vs. PC2, PC1 vs. PC3, and PC2 vs. PC3. **(C)** Cluster dendrogram depicting the relatedness of samples based on their global gene expression profiles. **(D)** Gene body coverage plot (output by RSeQC), showing the normalized read coverage across the length of genes (from 5’ to 3’ end) for each sample, which assesses potential 5’ or 3’ bias. **(E)** Gene body coverage heatmap (output by RSeQC), providing an alternative visualization of the uniformity of read coverage across gene bodies for all samples.
(PDF)

S7 Fig Quality control (QC) metrics for the ATAC-seq data of the GSE261119. **(A)** Bar plot showing the ATAC-seq read alignment rates for all samples. **(B)** Summary table of key ATAC-seq QC metrics generated by MultiQC, including duplication rate (Dups), GC content, average and median read length, failure rate, and total number of sequences for each sample. **(C)** Three-dimensional Principal Component Analysis (3D PCA) plots showing epigenomic sample relationships and variance across pairs of principal components (PC1 vs. PC2, PC2 vs. PC3). **(D)** Cluster dendrogram depicting the relatedness of samples based on their chromatin accessibility profiles. **(E)** Transcription start site (TSS) enrichment plot (output by ATACseqQC) for sample SRR28263042, indicating the quality of the ATAC-seq library by the nucleosome-free signal at gene promoters. **(F)** Transcription start site (TSS) enrichment heatmap (output by ATACseqQC) for sample SRR28263042, providing an alternative visualization of the enrichment signal.
(PDF)

S8 Fig Genomic annotation of ATAC-seq peaks and identification of shared genes in the GSE261119. **(A)** Bar plot showing the total number of ATAC-seq peaks annotated to various genomic regions (e.g., promoter, enhancer, intron, intergenic). **(B)** Venn diagram showing the overlap between genes with significantly down-regulated expression (RNA-seq, log2FC < −0.2, p-value < 0.05) and genes associated with significantly down-regulated ATAC-seq peaks. **(C)** Volcano plot displaying the differential expression of all the shared genes associated with promoter region. Significantly co-upregulated and co-downregulated genes are highlighted. **(D)** Volcano plot displaying the differential expression of all the shared genes associated with enhancer region. Significantly co-upregulated and co-downregulated genes are highlighted.
(PDF)

S9 Fig Gene Set Enrichment Analysis (GSEA) of shared genes in the GSE261119. **(A, B)** GSEA result of Gene Ontology (GO) biological pathways significantly enriched for co-upregulated and co-downregulated genes associated with promoter regions, respectively. **(C, D)** GSEA result of GO biological pathways significantly enriched for co-upregulated and co-downregulated genes associated with enhancer regions, respectively. **(E)** Representative GSEA plot for the GO term GO:0009165 (nucleotide biosynthetic process) enriched in promoter-associated co-upregulated genes. **(F)** Representative GSEA plot for the GO term GO:0042127 (regulation of cell population proliferation) enriched in promoter-associated co-downregulated genes.
(PDF)

S10 Fig Transcription factor (TF) analysis and validation of key regulators in the GSE261119. **(A)** Heatmap of RNA-seq expression levels for TFs whose binding motifs are significantly enriched in the promoters of co-downregulated genes and in the enhancers of both co-upregulated and co-downregulated genes. Genes related to the original research are highlighted. **(B)** Integrative Genomics Viewer (IGV) browser tracks showing increased chromatin accessibility (ATAC-seq) at the genomic locus of ETV4, which is also up-regulated at the RNA-seq level. **(C)** Integrative Genomics Viewer (IGV) browser tracks showing decreased chromatin accessibility (ATAC-seq) at the genomic locus of ETS1, which is also down-regulated at the RNA-seq level.
(PDF)

S11 Fig Custom analysis of an erythropoiesis and MAPK signaling-related gene set from the GSE261119 study. **(A, B)** Gene Set Enrichment Analysis (GSEA) result of significantly enriched biological pathways (|NES| > 1, p-value < 0.05) for the user-provided custom gene set of 92 erythropoiesis and MAPK signaling-related genes. **(C)** Heatmap of RNA-seq expression levels for the transcription factors (TFs) with binding motifs significantly enriched in the promoter regions of the custom gene set. **(D)** Network graph of the top significantly enriched transcription factors (TFs) for the custom genes, where node size represents the enrichment score and node color indicates statistical significance.
(PDF)

S12 Fig Local UI for the RAGER Snakemake Pipeline. **(A)** RAGER Snakemake Pipeline UI homepage. (B) Text-based results generated by the UI. **(C)** Graphical results generated by the UI.
(PDF)
