# Correction: RAGER: A user-friendly computational platform for integrated analysis of RNA-Seq and ATAC-seq data

**DOI:** 10.1371/journal.pone.0357056

**Published:** 2026-08-26

**Authors:** Yunjie Liu, Yijia Liu, Zhouming Zhang, Fei Li, Hua Yu, Lu Lu

There are errors in the author affiliations. The correct affiliations are as follows:

Yunjie Liu^1,2^, Yijia Liu^3^, Zhouming Zhang^4^, Fei Li^5^, Hua Yu^2,6^, Lu Lu^1^

**1** School of Intelligent Medicine and Information Engineering, Jiangxi University of Chinese Medicine, 330004, China, **2** Jiangxi Provincial Key Laboratory of Tumor Biology, School of Basic Medicine; Jiangxi Medical College, Nanchang University, 330031, China, **3** The First School of Clinical Medicine of Nanchang University, 330031, China, **4** Queen Mary School, Nanchang University, 330031, China, **5** Jiangxi Provincial Key Laboratory of Hematological Diseases, Department of Hematology, The First Affiliated Hospital, Jiangxi Medical College, Nanchang University, 330031, China, **6** The MOE Basic Research and Innovation Center for the Targeted Therapeutics of Solid Tumors, School of Basic Medicine; The First Affiliated Hospital, Jiangxi Medical College, Nanchang University, Nanchang, Jiangxi, 330031, China.
