# Supplementary material for: RAGER: A user-friendly computational platform for integrated analysis of RNA-Seq and ATAC-seq data
Source: PLoS One. 2026 May 22;21(5):e0349941. doi: 10.1371/journal.pone.0349941 (PMC13196991; doi:10.1371/journal.pone.0349941)

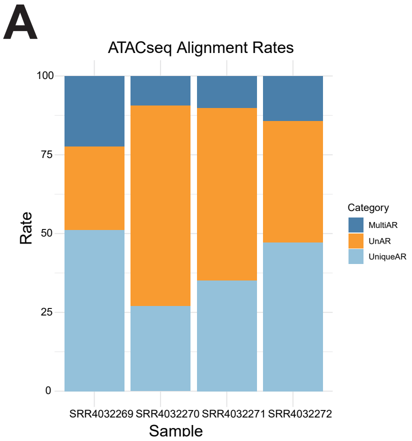

B

Sample Name	Dups	GC	Avg len	Median len	Failed	Seqs
**SRR4032269_1_val_1**	48.70%	45.00%	107bp	124bp	9%	29.0M
**SRR4032269_2_val_2**	48.60%	45.00%	107bp	124bp	0%	29.0M
**SRR4032270_1_val_1**	36.30%	48.00%	94bp	98bp	9%	28.9M
**SRR4032270_2_val_2**	36.60%	48.00%	94bp	98bp	0%	28.9M
**SRR4032271_1_val_1**	28.00%	47.00%	100bp	116bp	0%	22.3M
**SRR4032271_2_val_2**	27.90%	47.00%	100bp	116bp	0%	22.3M
**SRR4032272_1_val_1**	29.60%	47.00%	101bp	118bp	9%	24.5M
**SRR4032272_2_val_2**	30.00%	47.00%	101bp	118bp	0%	24.5M

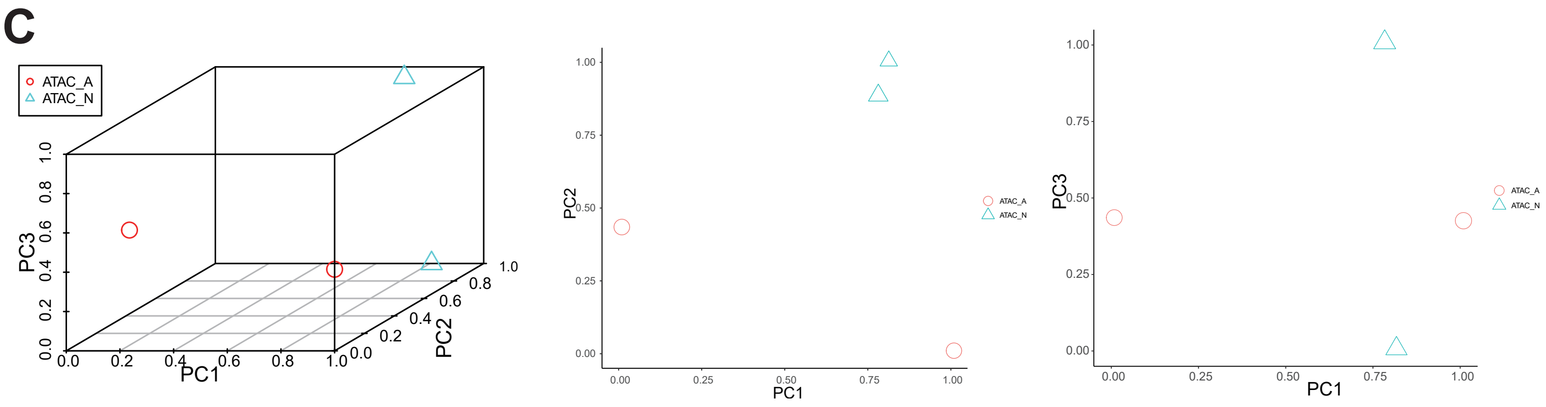

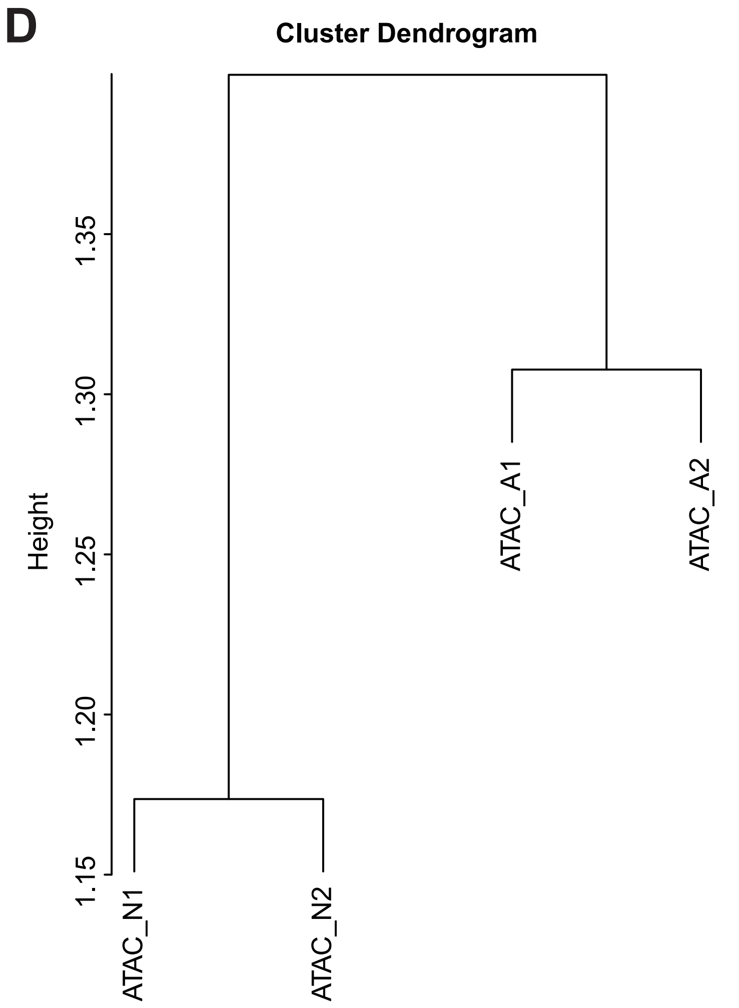

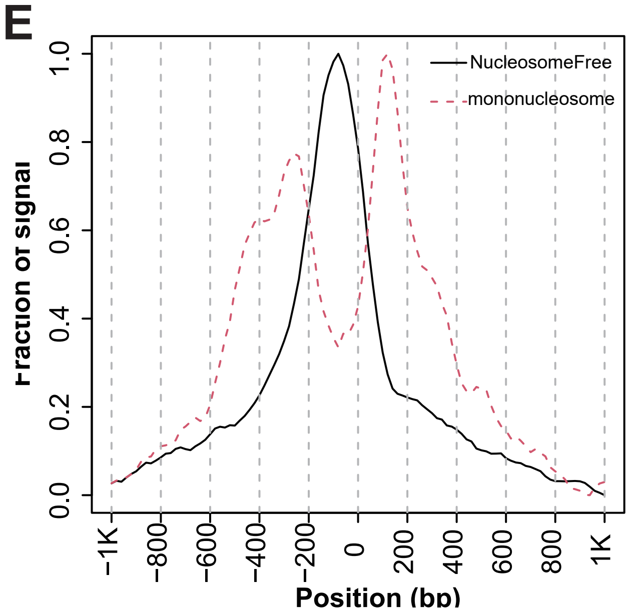

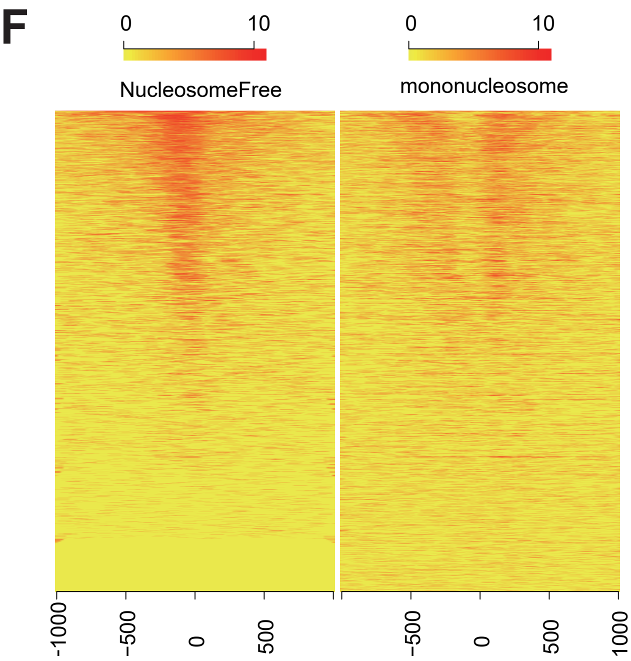

Supplement: S2 Fig — (A) Bar plot showing the ATAC-seq read alignment rates for all samples. (B) Summary table of key ATAC-seq QC metrics generated by MultiQC, including duplication rate (Dups), GC content, average and median read length, failure rate, and total number of sequences for each sample. (C) Three-dimensional Principal Component Analysis (3D PCA) plots showing epigenomic sample relationships and variance across pairs of principal components (PC1 vs. PC2, PC1 vs. PC3). (D) Cluster dendrogram depicting the relatedness of samples based on their chromatin accessibility profiles. (E) Transcription start site (TSS) enrichment plot (output by ATACseqQC) for sample SRR4032269, indicating the quality of the ATAC-seq library by the nucleosome-free signal at gene promoters. (F) Transcription start site (TSS) enrichment heatmap (output by ATACseqQC) for sample SRR4032269, providing an alternative visualization of the enrichment signal. (PDF) [file pone.0349941.s002.pdf]
