# Supplementary figures and images for: RAGER: A user-friendly computational platform for integrated analysis of RNA-Seq and ATAC-seq data
Source: PLoS One. 2026 May 22;21(5):e0349941. doi: 10.1371/journal.pone.0349941 (PMC13196991; doi:10.1371/journal.pone.0349941)

**A**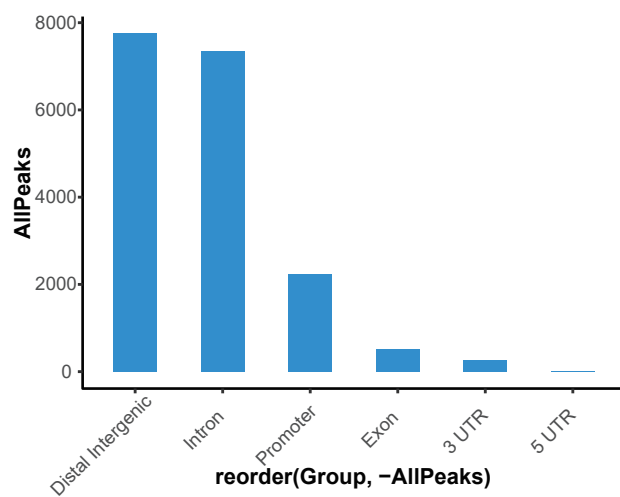**B**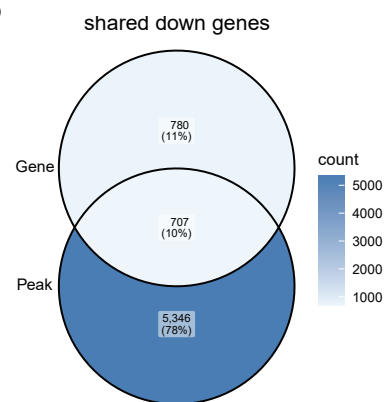**C**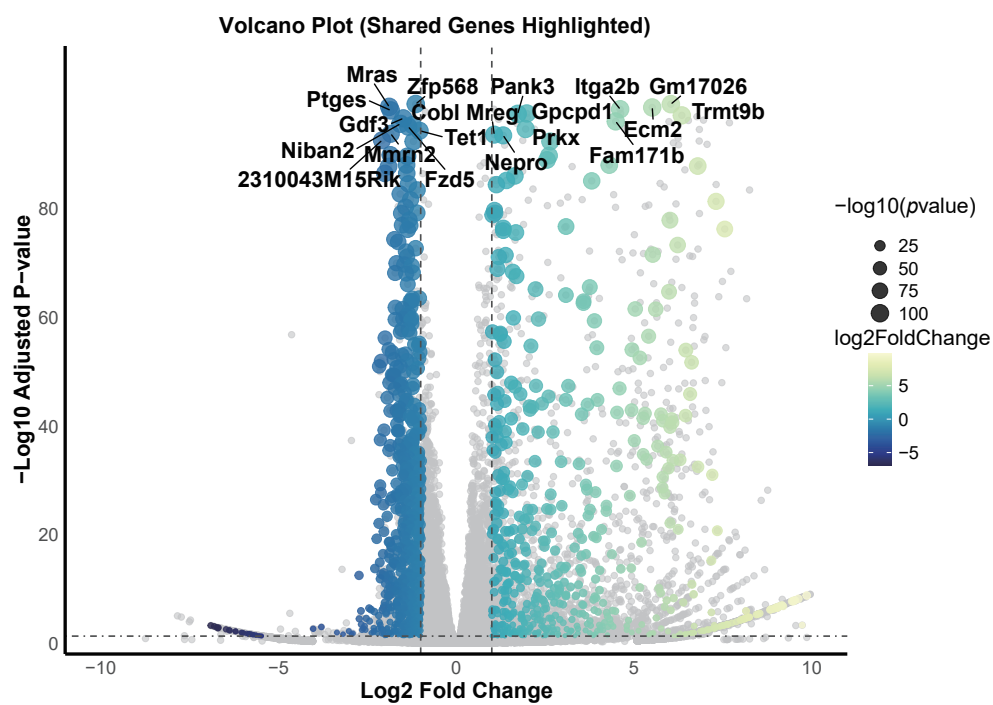**D**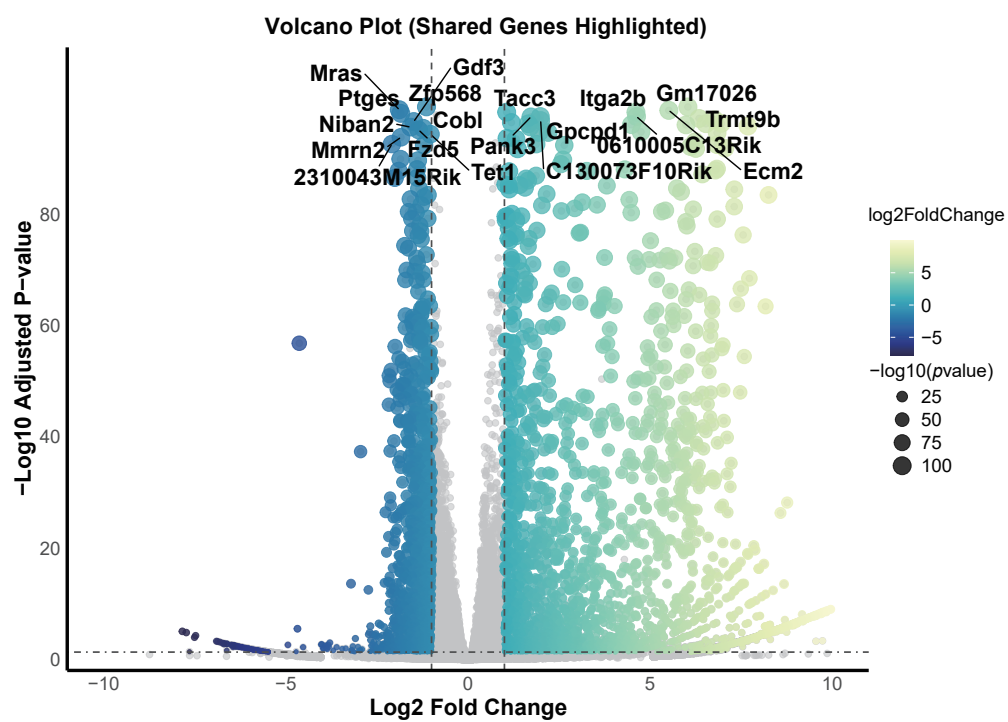

Supplement: S3 Fig — (A) Bar plot showing the total number of ATAC-seq peaks annotated to various genomic regions (e.g., promoter, enhancer, intron, intergenic). (B) Venn diagram showing the overlap between genes with significantly down-regulated expression (RNA-seq, log2FC < −1, p-value < 0.05) and genes associated with significantly down-regulated ATAC-seq peaks. (C) Volcano plot displaying the differential expression of all the shared genes associated with promoter region. Significantly co-upregulated and co-downregulated genes are highlighted. (D) Volcano plot displaying the differential expression of all the shared genes associated with enhancer region. Significantly co-upregulated and co-downregulated genes are highlighted. (PDF) [file pone.0349941.s003.pdf]

A

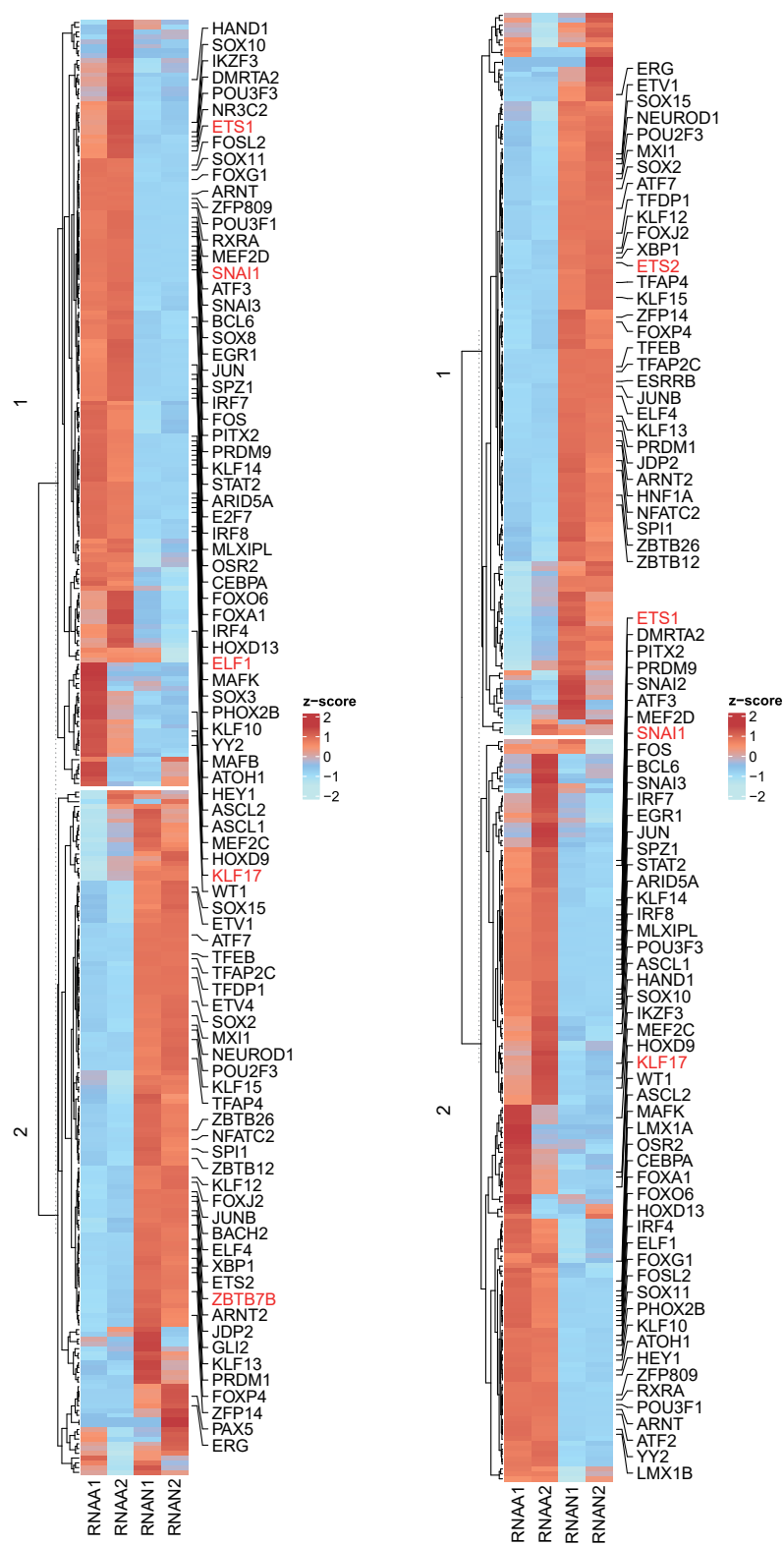

B

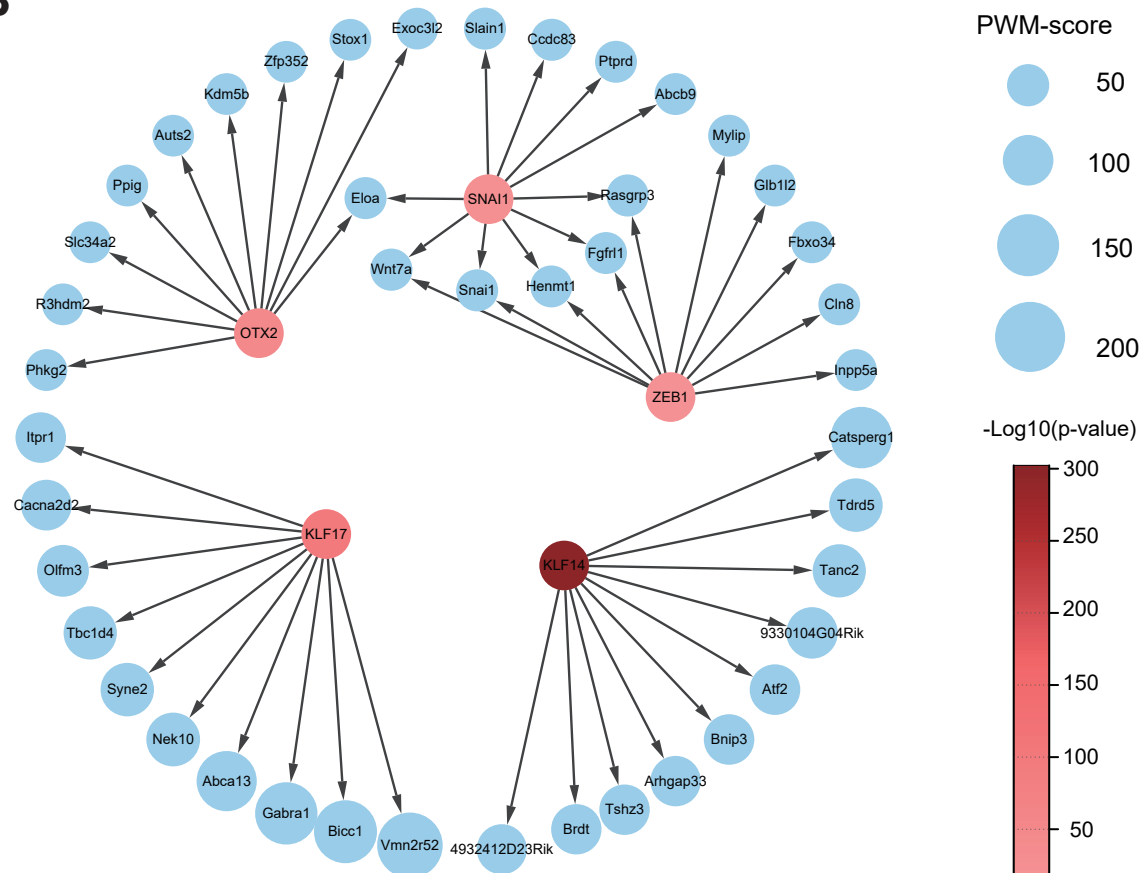

C

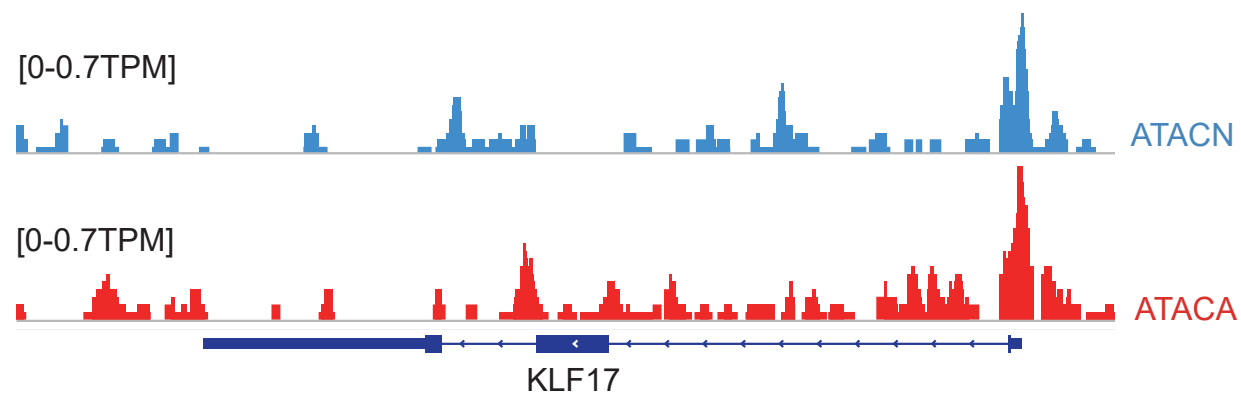

Supplement: S5 Fig — (A) Heatmap of RNA-seq expression levels for TFs whose binding motifs are significantly enriched in the co-upregulated and co-downregulated enhancer regions. Genes related to the original research are highlighted. (B) Cytoscape network graph depicting the top 10 target genes (ranked by enrichment score) for each transcription factor (TF), with regulatory relationships validated by the original research (TFs were significantly enriched to shared up-regulated genes associated with promoter regions). (C) Integrative Genomics Viewer (IGV) browser tracks showing increased chromatin accessibility (ATAC-seq) at the genomic locus of KLF17, which is also up-regulated at the RNA-seq level. (PDF) [file pone.0349941.s012.pdf]

**A**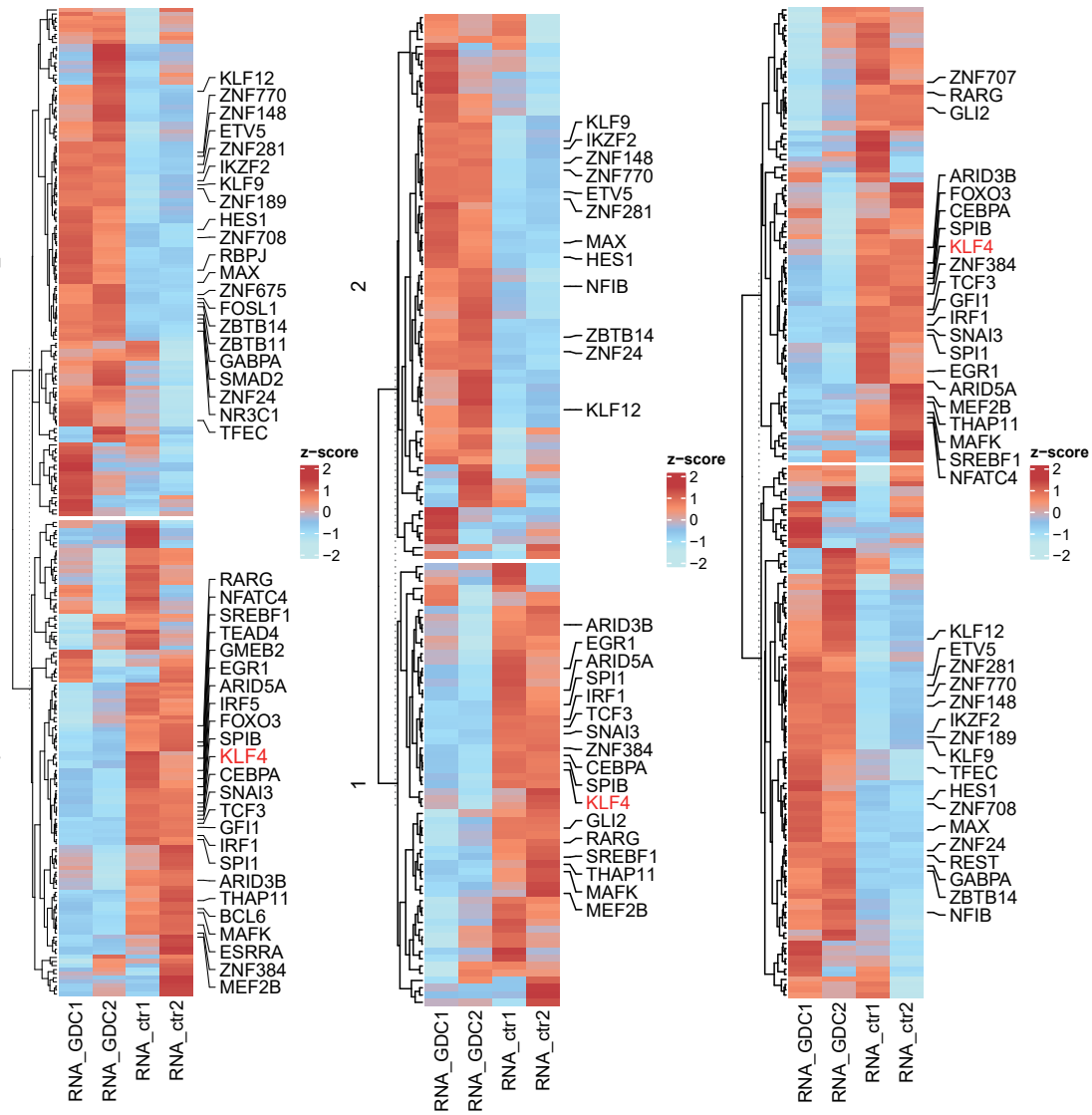**B**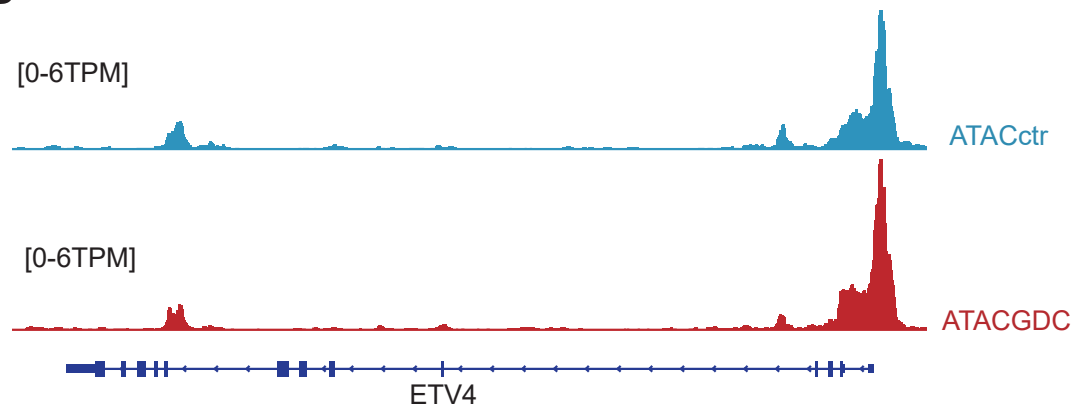**C**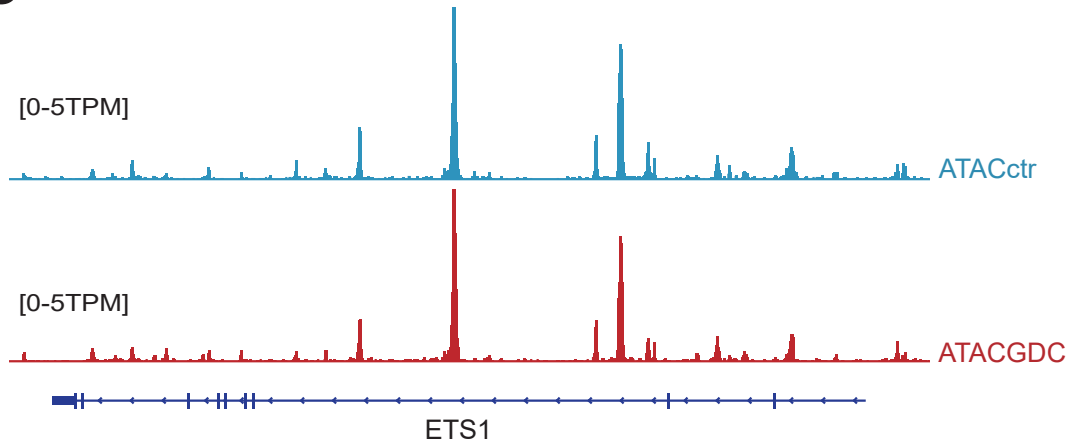

Supplement: S10 Fig — (A) Heatmap of RNA-seq expression levels for TFs whose binding motifs are significantly enriched in the promoters of co-downregulated genes and in the enhancers of both co-upregulated and co-downregulated genes. Genes related to the original research are highlighted. (B) Integrative Genomics Viewer (IGV) browser tracks showing increased chromatin accessibility (ATAC-seq) at the genomic locus of ETV4, which is also up-regulated at the RNA-seq level. (C) Integrative Genomics Viewer (IGV) browser tracks showing decreased chromatin accessibility (ATAC-seq) at the genomic locus of ETS1, which is also down-regulated at the RNA-seq level. (PDF) [file pone.0349941.s009.pdf]
