# Supplementary material for: RAGER: A user-friendly computational platform for integrated analysis of RNA-Seq and ATAC-seq data
Source: PLoS One. 2026 May 22;21(5):e0349941. doi: 10.1371/journal.pone.0349941 (PMC13196991; doi:10.1371/journal.pone.0349941)

A

Description	NES	pvalue
signaling receptor activity	1.619189852	$2.57*10^{-4}$
kinase regulator activity	1.703126929	$1.79*10^{-2}$
nuclear envelope	1.649271106	$1.14*10^{-2}$
G protein-coupled receptor activity	1.5706388	$1.23*10^{-2}$
G protein-coupled receptor signaling pathway	1.347890471	$3.50*10^{-2}$

B

Description	NES	pvalue
positive regulation of cell differentiation	-1.04523	$5*10^{-2}$
cellular process involved in reproduction in multicellular organism	-1.0406	$4.20*10^{-2}$
positive regulation of protein phosphorylation	-1.04258	$4.70*10^{-2}$
regulation of cell-substrate adhesion	-1.02831	$3.20*10^{-2}$
positive regulation of RNA metabolic process	-1.00114	$3.31*10^{-3}$

C

Description	NES	pvalue
translation initiation factor activity	1.71	$3.09*10^{-3}$
translation regulator activity	1.57	$1.04*10^{-2}$
translation regulator activity, nucleic acid binding	1.55	$2.06*10^{-2}$
Cul2-RING ubiquitin ligase complex	1.68	$3.24*10^{-3}$
ubiquitin ligase-substrate adaptor activity	1.61	$5.17*10^{-3}$

D

Description	NES	pvalue
positive regulation of intracellular signal transduction	-1.05606	$3.60*10^{-2}$
positive regulation of small GTPase mediated signal transduction	-1.04392	$2.50*10^{-2}$
positive regulation of response to stimulus	-1.04377	$2.15*10^{-2}$
positive regulation of phosphorylation	-1.03928	$1.41*10^{-2}$
negative regulation of cellular component organization	-1.05086	$3.40*10^{-2}$

E

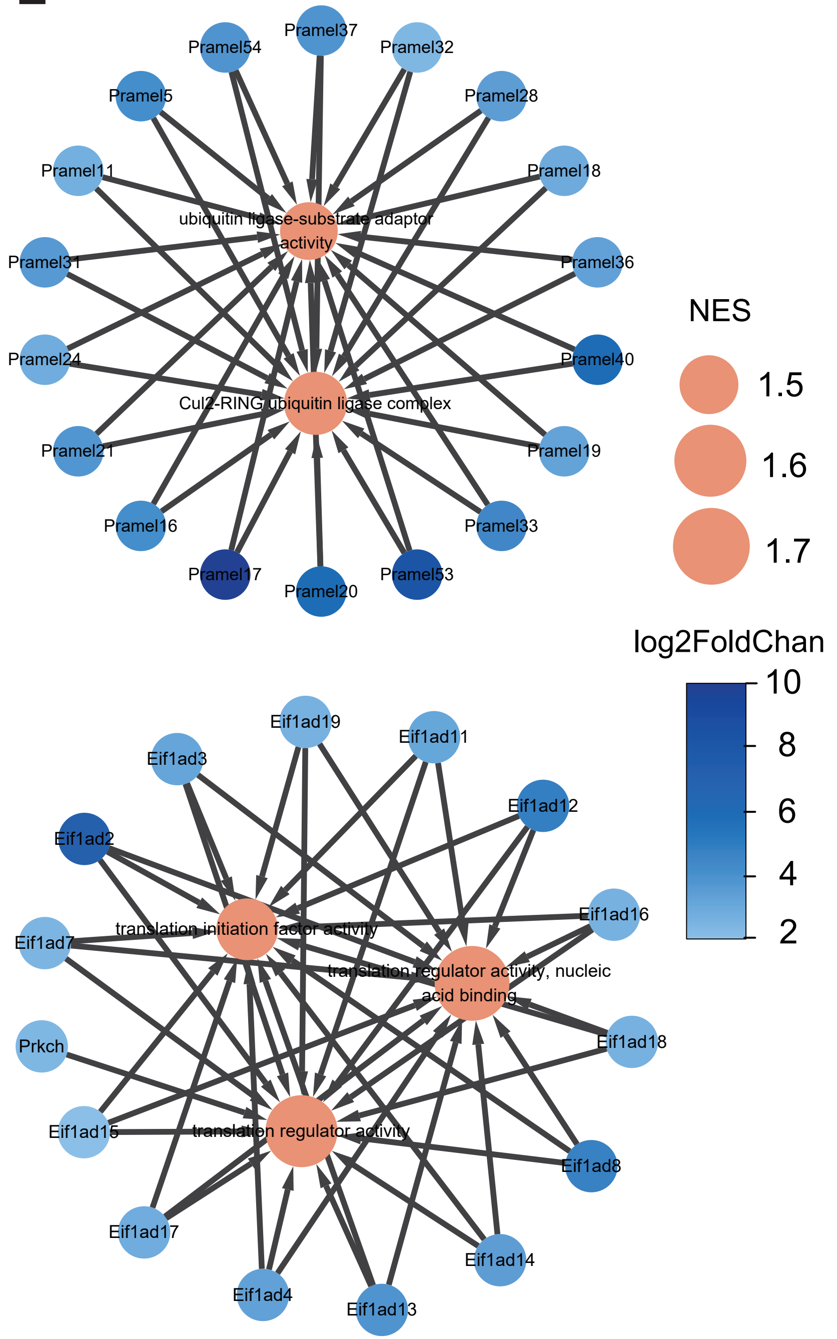

Supplement: S4 Fig — (A, B) GSEA result of Gene Ontology (GO) biological pathways significantly enriched (|NES| > 1, p-value < 0.05) for co-upregulated and co-downregulated genes associated with promoter regions, respectively. (C, D) GSEA result of GO biological pathways significantly enriched (|NES| > 1, p-value < 0.05) for co-upregulated and co-downregulated genes associated with enhancer regions, respectively. (E) Cytoscape network visualization of GO biological pathways significantly enriched (|NES| > 1, p-value < 0.05) for the shared up-regulated genes associated with enhancer regions. (PDF) [file pone.0349941.s004.pdf]
