# Supplementary material for: RAGER: A user-friendly computational platform for integrated analysis of RNA-Seq and ATAC-seq data
Source: PLoS One. 2026 May 22;21(5):e0349941. doi: 10.1371/journal.pone.0349941 (PMC13196991; doi:10.1371/journal.pone.0349941)

A

Sample Name	Dups	GC	Avg len	Median len	Failed	Seqs
SRR28264346_1_val_1	59.10%	52.00%	149bp	150bp	18%	26.9M
SRR28264346_2_val_2	58.40%	52.00%	149bp	150bp	18%	26.9M
SRR28264347_1_val_1	50.70%	52.00%	149bp	150bp	18%	20.3M
SRR28264347_2_val_2	50.60%	52.00%	149bp	150bp	9%	20.3M
SRR28264348_1_val_1	62.70%	54.00%	150bp	150bp	9%	29.0M
SRR28264348_2_val_2	61.60%	54.00%	150bp	150bp	9%	29.0M
SRR28264349_1_val_1	55.00%	53.00%	150bp	150bp	9%	22.7M
SRR28264349_2_val_2	53.60%	53.00%	150bp	150bp	9%	22.7M

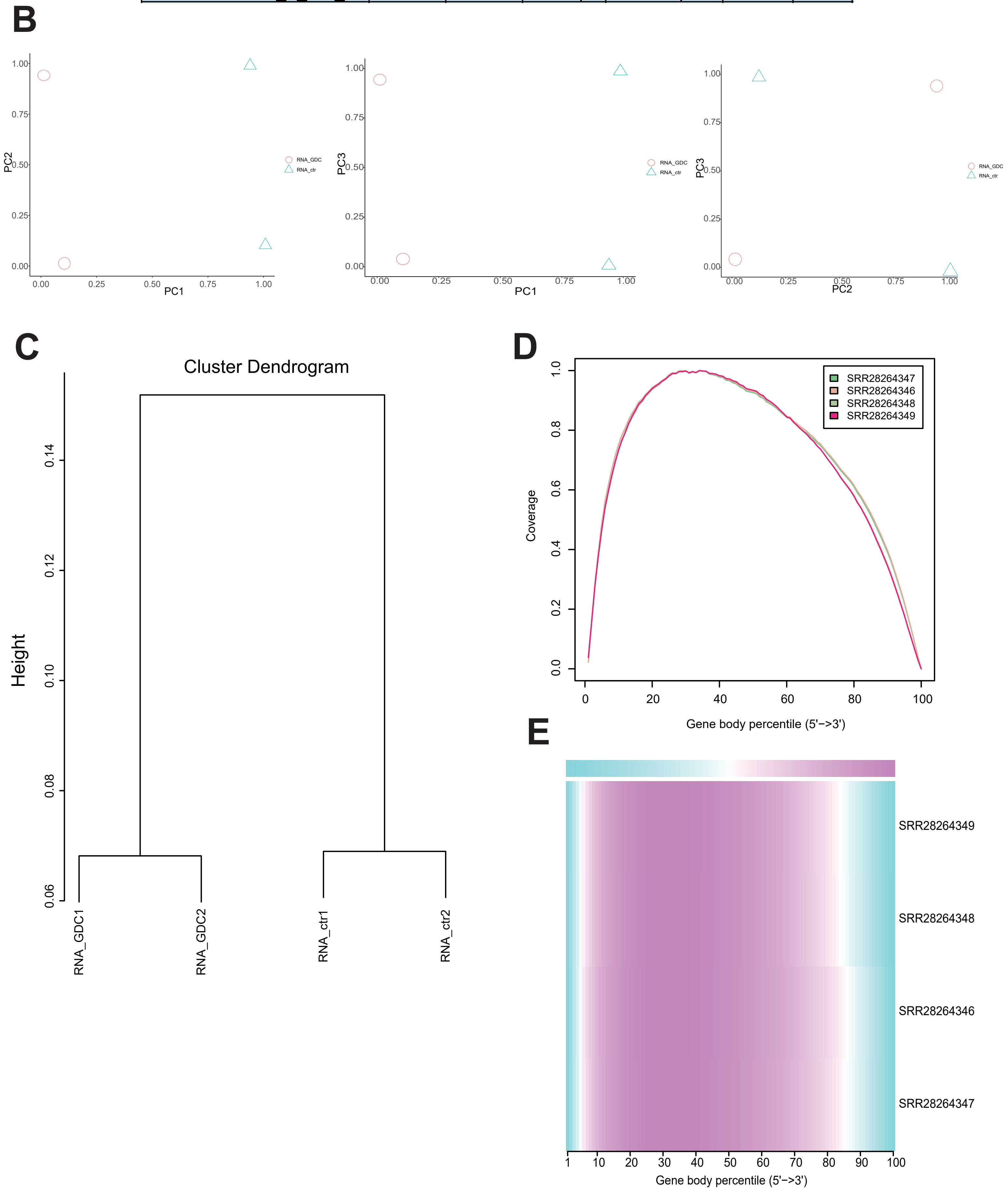

Supplement: S6 Fig — (A) Summary table of key QC metrics generated by MultiQC, including duplication rate (Dups), GC content, average and median read length, failure rate, and total number of sequences for each sample. (B) Principal Component Analysis (PCA) plots showing sample relationships and variance across three pairs of principal components: PC1 vs. PC2, PC1 vs. PC3, and PC2 vs. PC3. (C) Cluster dendrogram depicting the relatedness of samples based on their global gene expression profiles. (D) Gene body coverage plot (output by RSeQC), showing the normalized read coverage across the length of genes (from 5’ to 3’ end) for each sample, which assesses potential 5’ or 3’ bias. (E) Gene body coverage heatmap (output by RSeQC), providing an alternative visualization of the uniformity of read coverage across gene bodies for all samples. (PDF) [file pone.0349941.s005.pdf]
