# Supplementary material for: RAGER: A user-friendly computational platform for integrated analysis of RNA-Seq and ATAC-seq data
Source: PLoS One. 2026 May 22;21(5):e0349941. doi: 10.1371/journal.pone.0349941 (PMC13196991; doi:10.1371/journal.pone.0349941)

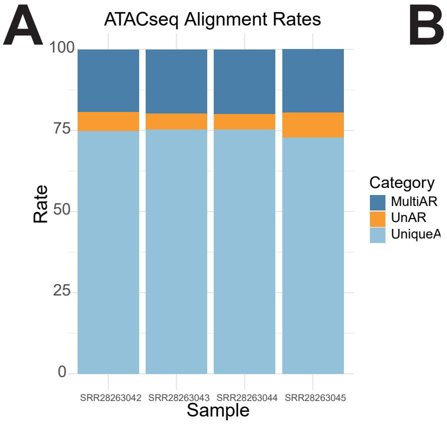

B

Sample Name	Dups	GC	Avg len	Median len	Failed	Seqs
**SRR28263042_1_val_1**	38.20%	53.00%	119bp	150bp	9%	34.4M
**SRR28263042_2_val_2**	38.70%	53.00%	119bp	150bp	9%	34.4M
**SRR28263043_1_val_1**	41.00%	53.00%	118bp	150bp	9%	39.6M
**SRR28263043_2_val_2**	40.50%	53.00%	118bp	147bp	9%	39.6M
**SRR28263044_1_val_1**	36.50%	53.00%	116bp	147bp	9%	37.0M
**SRR28263044_2_val_2**	38.20%	53.00%	116bp	147bp	9%	37.0M
**SRR28263045_1_val_1**	38.20%	53.00%	117bp	150bp	9%	34.8M
**SRR28263045_2_val_2**	37.40%	53.00%	117bp	147bp	9%	34.8M

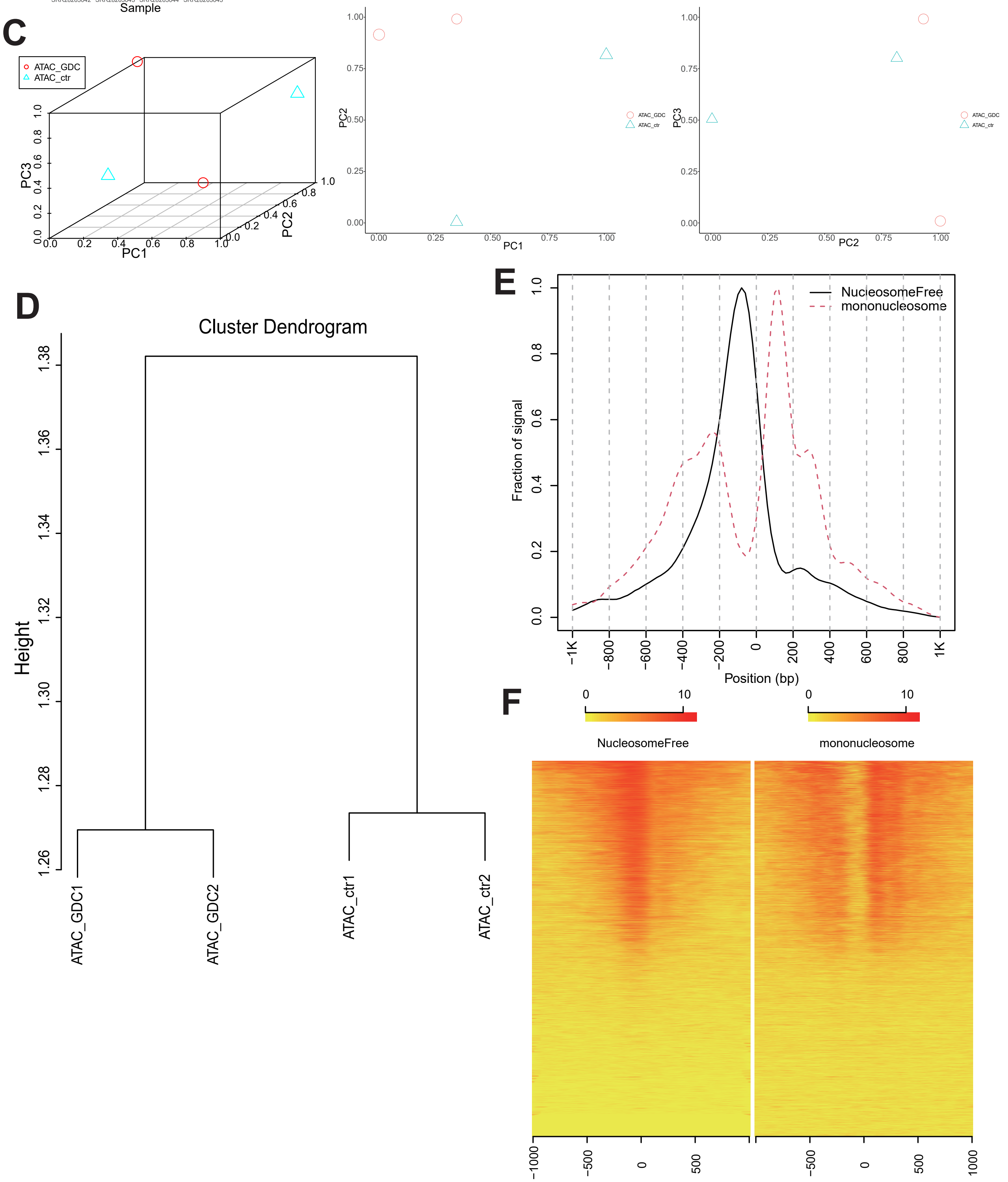

Supplement: S7 Fig — (A) Bar plot showing the ATAC-seq read alignment rates for all samples. (B) Summary table of key ATAC-seq QC metrics generated by MultiQC, including duplication rate (Dups), GC content, average and median read length, failure rate, and total number of sequences for each sample. (C) Three-dimensional Principal Component Analysis (3D PCA) plots showing epigenomic sample relationships and variance across pairs of principal components (PC1 vs. PC2, PC2 vs. PC3). (D) Cluster dendrogram depicting the relatedness of samples based on their chromatin accessibility profiles. (E) Transcription start site (TSS) enrichment plot (output by ATACseqQC) for sample SRR28263042, indicating the quality of the ATAC-seq library by the nucleosome-free signal at gene promoters. (F) Transcription start site (TSS) enrichment heatmap (output by ATACseqQC) for sample SRR28263042, providing an alternative visualization of the enrichment signal. (PDF) [file pone.0349941.s006.pdf]
