# Supplementary material for: RAGER: A user-friendly computational platform for integrated analysis of RNA-Seq and ATAC-seq data
Source: PLoS One. 2026 May 22;21(5):e0349941. doi: 10.1371/journal.pone.0349941 (PMC13196991; doi:10.1371/journal.pone.0349941)

A

Description	NES	pvalue
energy derivation by oxidation of organic compounds	1.778059572	$3.90*10^{-2}$
nucleotide biosynthetic process	1.703264454	$4.60*10^{-2}$
positive regulation of multicellular organismal process	1.702587819	$1.55*10^{-2}$
mitochondrial membrane	1.801778915	$7.55*10^{-3}$
response to radiation	1.893627305	$7.40*10^{-3}$

B

Description	NES	pvalue
positive regulation of cell population proliferation	-1.71559	$2.90*10^{-2}$
regulation of cell population proliferation	-1.64877	$4.32*10^{-2}$
regulation of cell-cell adhesion	-1.96903	$3.90*10^{-3}$
G protein-coupled receptor activity	-1.77422	$4.10*10^{-2}$
regulation of immune response	-1.6459	$4.30*10^{-2}$

C

Description	NES	pvalue
positive regulation of neuron projection development	1.887540347	$4.50*10^{-2}$

D

Description	NES	pvalue
translation	-1.64625	$3.80*10^{-2}$
peptide biosynthetic process	-1.58351	$5.00*10^{-2}$
positive regulation of translation	-2.13724	$1.41*10^{-2}$
T cell receptor signaling pathway	-2.05074	$9.79*10^{-3}$
antigen receptor-mediated signaling pathway	-1.7884	$4.50*10^{-2}$

E

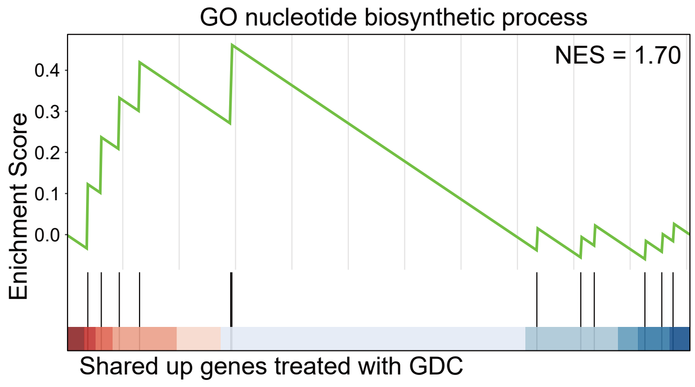

F

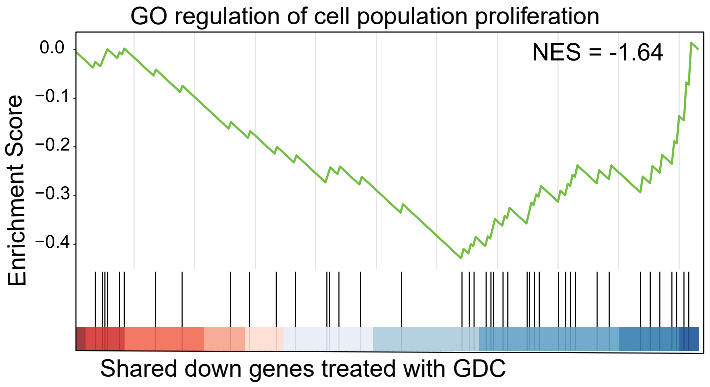

Supplement: S9 Fig — (A, B) GSEA result of Gene Ontology (GO) biological pathways significantly enriched for co-upregulated and co-downregulated genes associated with promoter regions, respectively. (C, D) GSEA result of GO biological pathways significantly enriched for co-upregulated and co-downregulated genes associated with enhancer regions, respectively. (E) Representative GSEA plot for the GO term GO:0009165 (nucleotide biosynthetic process) enriched in promoter-associated co-upregulated genes. (F) Representative GSEA plot for the GO term GO:0042127 (regulation of cell population proliferation) enriched in promoter-associated co-downregulated genes. (PDF) [file pone.0349941.s008.pdf]
