# Supplementary material for: RAGER: A user-friendly computational platform for integrated analysis of RNA-Seq and ATAC-seq data
Source: PLoS One. 2026 May 22;21(5):e0349941. doi: 10.1371/journal.pone.0349941 (PMC13196991; doi:10.1371/journal.pone.0349941)

A

Description	NES	pvalue
chemical homeostasis	-1.65173	$9.87*10^{-3}$
response to oxidative stress	1.646823093	$1.28*10^{-2}$

B

Description	NES	pvalue
Metabolic pathways	-1.22605	$0.2*10^{-1}$

C

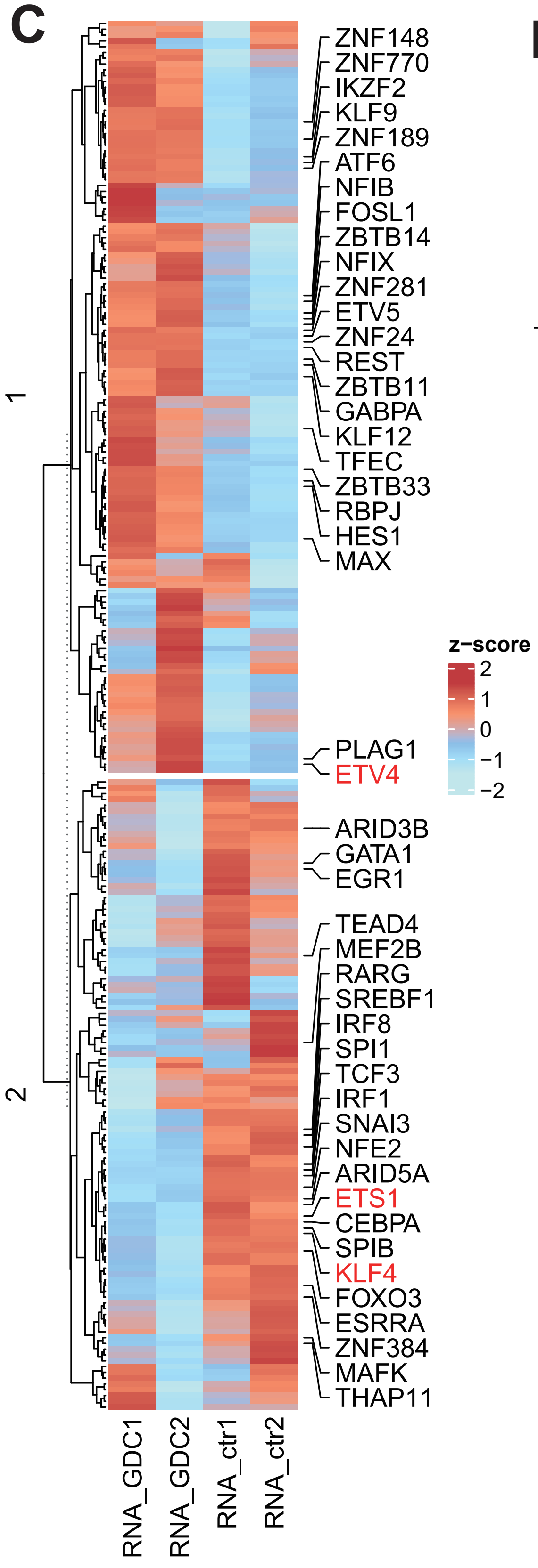

D

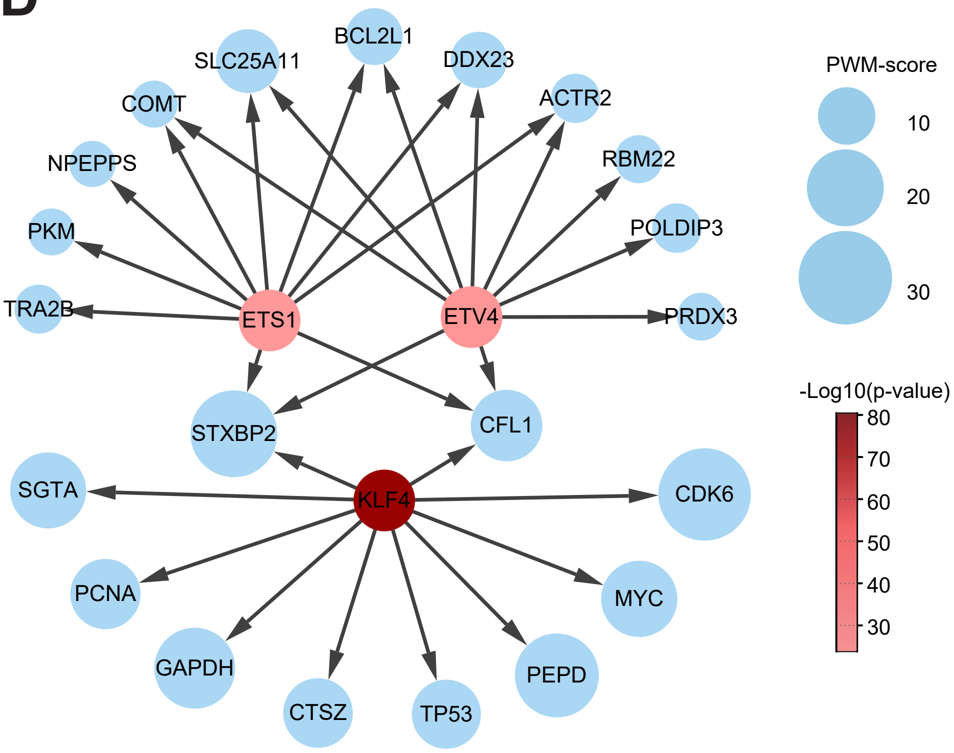

Supplement: S11 Fig — (A, B) Gene Set Enrichment Analysis (GSEA) result of significantly enriched biological pathways (|NES| > 1, p-value < 0.05) for the user-provided custom gene set of 92 erythropoiesis and MAPK signaling-related genes. (C) Heatmap of RNA-seq expression levels for the transcription factors (TFs) with binding motifs significantly enriched in the promoter regions of the custom gene set. (D) Network graph of the top significantly enriched transcription factors (TFs) for the custom genes, where node size represents the enrichment score and node color indicates statistical significance. (PDF) [file pone.0349941.s010.pdf]
