# Supplementary material for: RAGER: A user-friendly computational platform for integrated analysis of RNA-Seq and ATAC-seq data
Source: PLoS One. 2026 May 22;21(5):e0349941. doi: 10.1371/journal.pone.0349941 (PMC13196991; doi:10.1371/journal.pone.0349941)

A

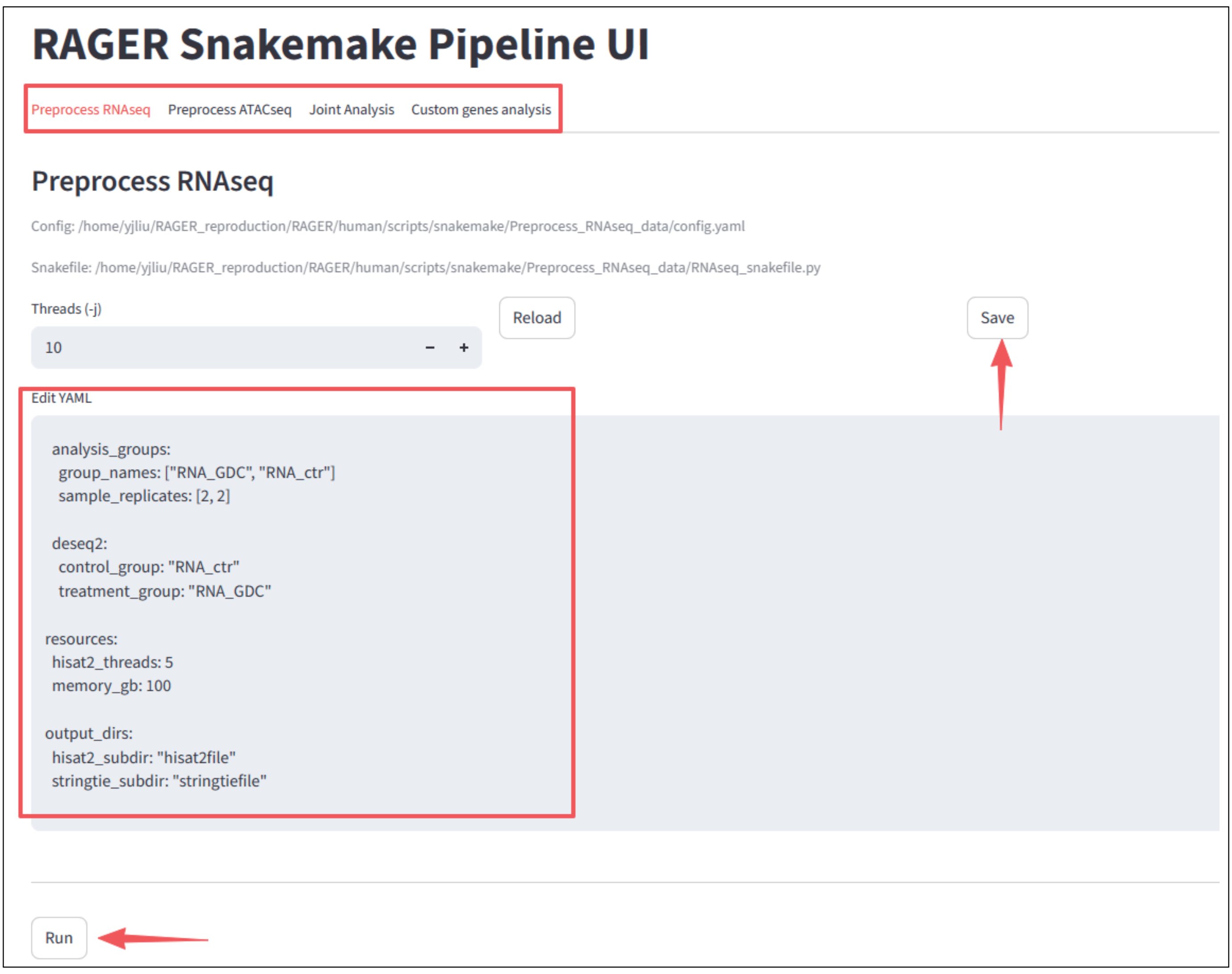

B

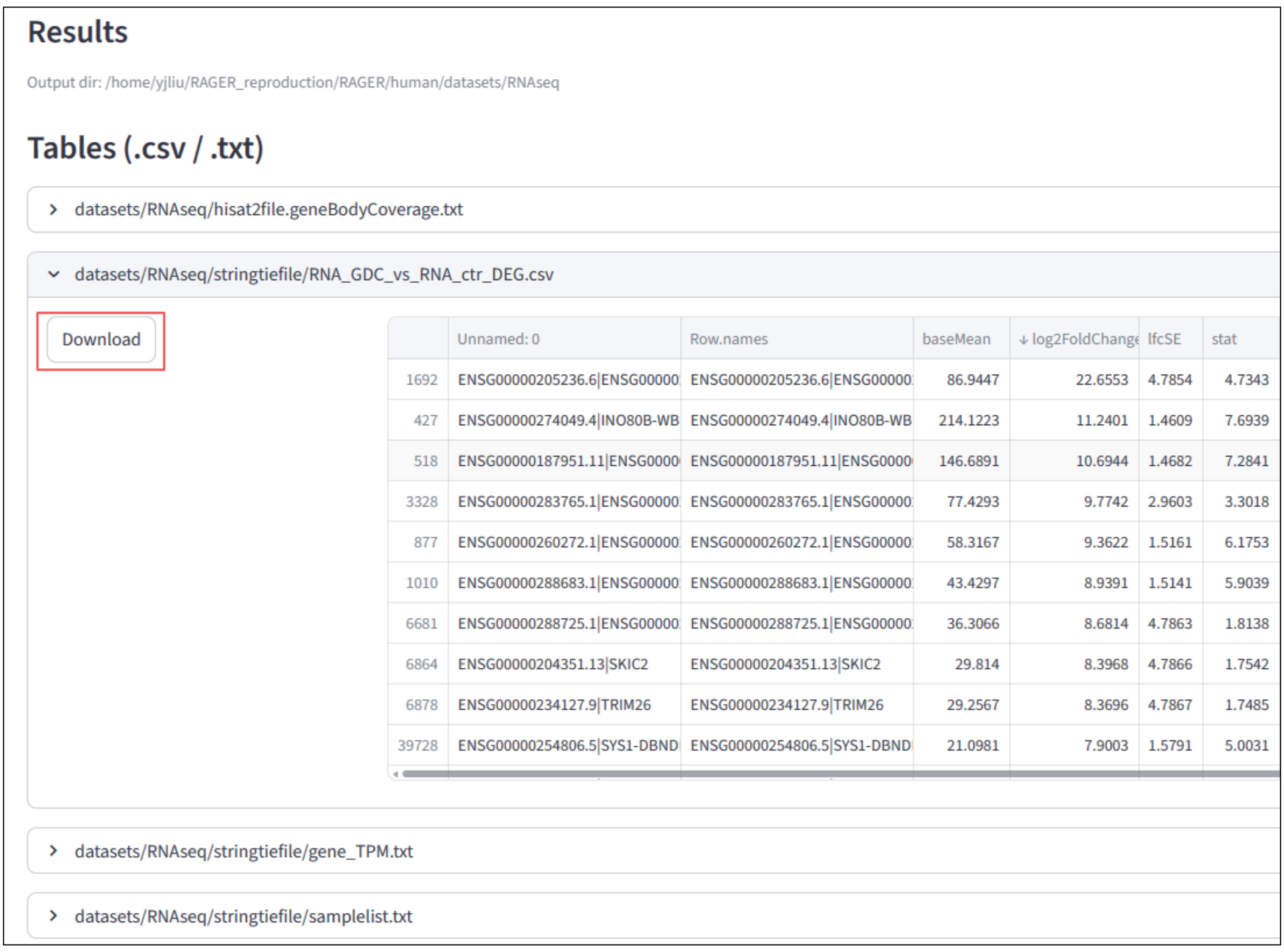

	Unnamed: 0	Row.names	baseMean	↓ log2FoldChange	lfcSE	stat
1692	ENSG00000205236.6|ENSG00000	ENSG00000205236.6|ENSG00000	86.9447	22.6553	4.7854	4.7343
427	ENSG00000274049.4|INO80B-WB	ENSG00000274049.4|INO80B-WB	214.1223	11.2401	1.4609	7.6939
518	ENSG00000187951.11|ENSG0000	ENSG00000187951.11|ENSG0000	146.6891	10.6944	1.4682	7.2841
3328	ENSG00000283765.1|ENSG00000	ENSG00000283765.1|ENSG00000	77.4293	9.7742	2.9603	3.3018
877	ENSG00000260272.1|ENSG00000	ENSG00000260272.1|ENSG00000	58.3167	9.3622	1.5161	6.1753
1010	ENSG00000288683.1|ENSG00000	ENSG00000288683.1|ENSG00000	43.4297	8.9391	1.5141	5.9039
6681	ENSG00000288725.1|ENSG00000	ENSG00000288725.1|ENSG00000	36.3066	8.6814	4.7863	1.8138
6864	ENSG00000204351.13|SKIC2	ENSG00000204351.13|SKIC2	29.814	8.3968	4.7866	1.7542
6878	ENSG00000234127.9|TRIM26	ENSG00000234127.9|TRIM26	29.2567	8.3696	4.7867	1.7485
39728	ENSG00000254806.5|SYS1-DBND	ENSG00000254806.5|SYS1-DBND	21.0981	7.9003	1.5791	5.0031

C

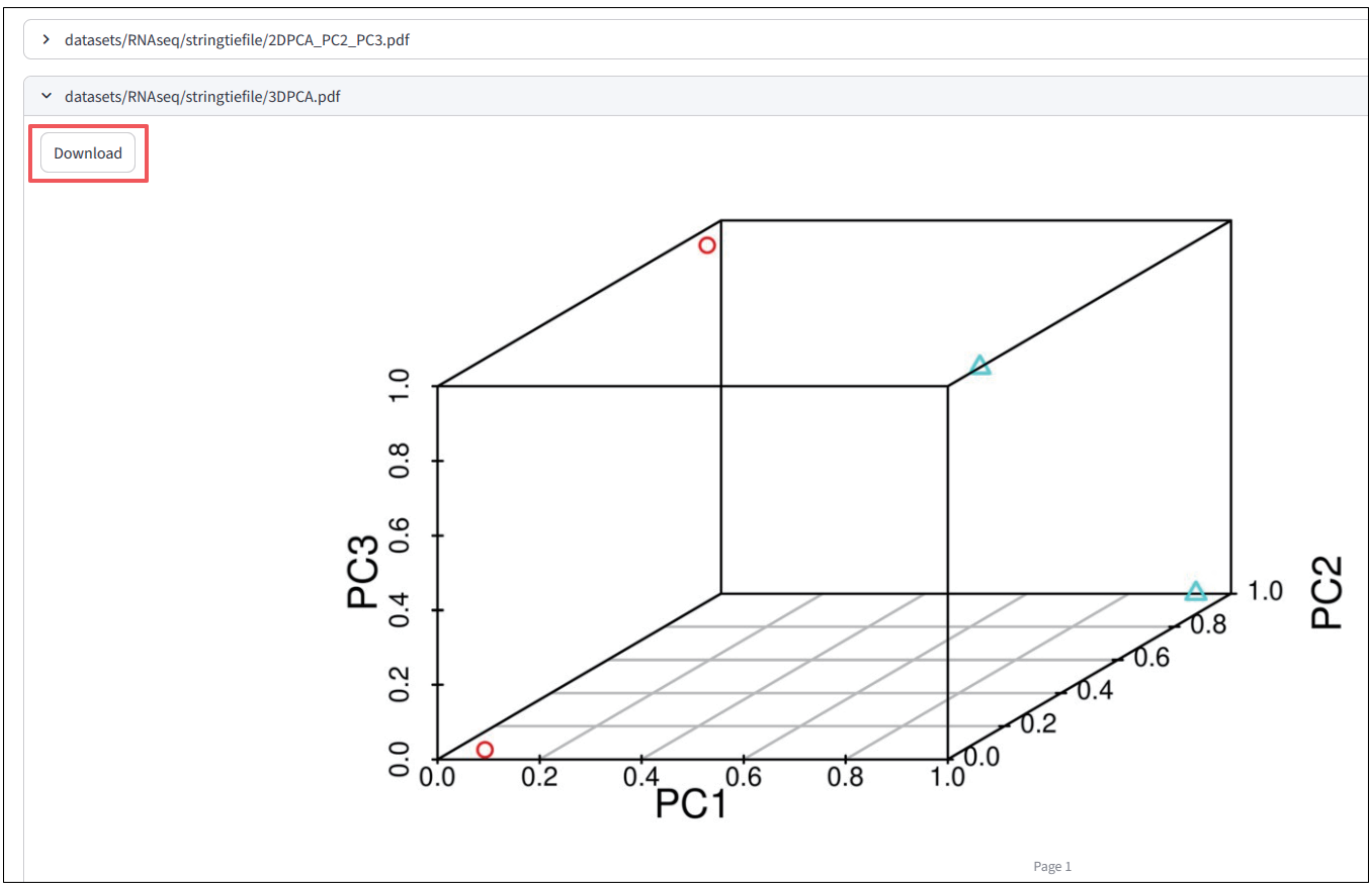

Supplement: S12 Fig — (A) RAGER Snakemake Pipeline UI homepage. (B) Text-based results generated by the UI. (C) Graphical results generated by the UI. (PDF) [file pone.0349941.s011.pdf]
