# Supplementary material for: RAGER: A user-friendly computational platform for integrated analysis of RNA-Seq and ATAC-seq data
Source: PLoS One. 2026 May 22;21(5):e0349941. doi: 10.1371/journal.pone.0349941 (PMC13196991; doi:10.1371/journal.pone.0349941)
Supplement: S8 Fig — (A) Bar plot showing the total number of ATAC-seq peaks annotated to various genomic regions (e.g., promoter, enhancer, intron, intergenic). (B) Venn diagram showing the overlap between genes with significantly down-regulated expression (RNA-seq, log2FC < −0.2, p-value < 0.05) and genes associated with significantly down-regulated ATAC-seq peaks. (C) Volcano plot displaying the differential expression of all the shared genes associated with promoter region. Significantly co-upregulated and co-downregulated genes are highlighted. (D) Volcano plot displaying the differential expression of all the shared genes associated with enhancer region. Significantly co-upregulated and co-downregulated genes are highlighted. (PDF) [file pone.0349941.s007.pdf]

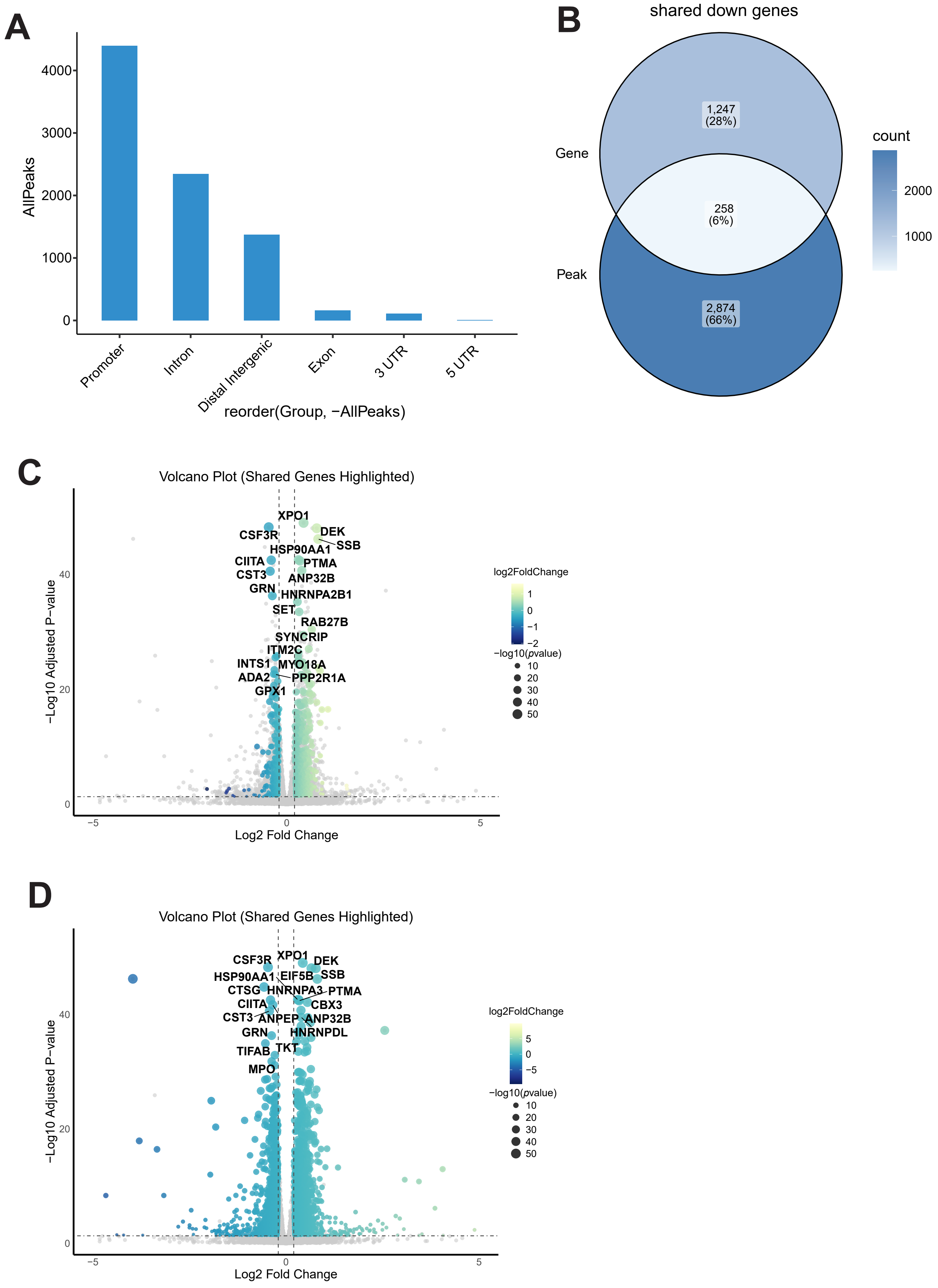

A
AllPeaks
4000
3000
2000
1000
0
Promoter
Intron
Distal Intergenic
Exon
3 UTR
5 UTR
reorder(Group, −AllPeaks)
B
shared down genes
Gene
Peak
1,247
(28%)
258
(6%)
2,874
(66%)
count
2000
1000
C
Volcano Plot (Shared Genes Highlighted)
XPO1
CSF3R
DEK
SSB
HSP90AA1
CIITA
PTMA
CST3
ANP32B
GRN
HNRNPA2B1
SET
RAB27B
SYNCRIP
ITM2C
INTS1
MYO18A
ADA2
PPP2R1A
GPX1
−Log10 Adjusted P−value
Log2 Fold Change
log2FoldChange
1
0
−1
−2
−log10(pvalue)
10
20
30
40
50
D
Volcano Plot (Shared Genes Highlighted)
CSF3R
XPO1
DEK
HSP90AA1
EIF5B
SSB
CTSG
HNRNPA3
PTMA
CIITA
CBX3
CST3
ANPEP
ANP32B
GRN
HNRNPDL
TIFAB
TKT
MPO
−Log10 Adjusted P−value
Log2 Fold Change
log2FoldChange
5
0
−5
−log10(pvalue)
10
20
30
40
50
